# RRLC-QTOF/MS-Based Metabolomics Reveal the Mechanism of Chemical Variations and Transformations of Astragali Radix as a Result of the Roasting Process

**DOI:** 10.3389/fchem.2022.903168

**Published:** 2022-05-05

**Authors:** Yang Li, Shenhui Huang, Jie Sun, Weiping Duan, Cunyu Li, Guoping Peng, Yunfeng Zheng

**Affiliations:** ^1^ Jiangsu Province Engineering Research Center of Classical Prescription, Nanjing University of Chinese Medicine, Nanjing, China; ^2^ Jiangsu Collaborative Innovation Center of Chinese Medicinal Resources Industrialization, Nanjing University of Chinese Medicine, Nanjing, China

**Keywords:** Astragali Radix, LC-QTOF/MS, non-targeted metabolomics, roasting process, malonyl isoflavonoids / cycloastragenols, acetyl isoflavonoids / cycloastragenols

## Abstract

Astragali Radix (AR), which is extensively used as a healthy food supplement and medicinal herb, contains two forms of products corresponding to raw Astragalus Radix (RAR) and processed Astragali Radix (PAR), which was obtained by roasting. In this study, a non-targeted rapid resolution liquid chromatography coupled with quadruple time-of-flight mass spectrometry (RRLC-Q/TOF-MS) based metabolomics approach was developed to investigate the chemical changes of AR due to roasting. A total of 63 compounds were identified or tentatively identified. Among them, 23 isoflavonoids (composed of 12 isoflavones, eight pterocarpans, and three isoflavans) and six cycloastragenols were characterized as differential metabolites. Heatmap visualization and high-performance liquid chromatography coupled with photodiode array and evaporative light scattering detector (HPLC–PDA-ELSD) quantitative analysis revealed that malonyl isoflavonoids or cycloastragenols were at higher levels in RAR. These might be converted to corresponding acetyl isoflavonoids and cycloastragenols and related isoflavonoid glycosides during roasting. To prove this prediction, chemical conversion experiments on malonyl isoflavonoids and cycloastragenols were performed to confirm and clarify the chemical transformation mechanism.

## Introduction

Astragali Radix (AR), a widely-known traditional food–medicinal herb has been widely utilized for the treatment of gastrointestinal diseases and immune regulation for hundreds of years in oriental countries (Li et al., 2011). Roasting is one of the most common processing methods in Chinese medicine that can improve the curative effect or eliminate side effects (Kang et al., 2022). Processed Astragali Radix (PAR), is obtained by roasting, considered to be more highly effective in tonifying the spleen and stomach in tradition Chinese medicine (TCM) (Pharmacopoeia of the People’s Republic of China, 2020). Chemical investigations have shown that isoflavonoids, triterpene saponins, and polysaccharides are the main active components of AR (Zhang et al., 2021). Modern pharmacological studies revealed that these active ingredients possess a variety of biological activities, including anti-diabetes, antiviral, anti-inflammatory, and in particular, immunomodulatory properties (Yin et al., 2004; Yakuboğulları et al., 2019; Zhang et al., 2019; Chen et al., 2020). Generally, ingredients of natural herbs may vary during the processing, leading to the changes in their quality and effectiveness. Therefore, it is critical to address unknowns related to the difference and variations of chemical composition of AR during the roasting process.

In practice, metabolomic analysis based on modern separation science has been developed as an effective tool for revealing the metabolites present and their levels under specific conditions at a given point in time (Zheng et al., 2020). Raw TCMs such as RAR are processed into different products in which the number of active metabolites varies because of the interaction of excipients and/or heating during processing. Therefore, interpretation of the mechanism of processed TCMs must discuss how the composition of active ingredients changes as a result of processing. It implies that by using metabolomics, it should be possible to comparatively evaluate the difference in TCMs before and after processing. However, the major challenge for the field of metabolomics is to detect and identify as many discriminating metabolites as possible within a single rapid analytical measurement. Rapid-resolution liquid chromatography combined with quadrupole time-of-flight mass spectrometry (RRLC-Q/TOF-MS) has become preferred analytical choices in plant metabolomics research because of their unsurpassed sensitivities and high resolutions (Klevorn and Dean, 2017; Chai et al., 2019). Previous studies have elucidated the composition variations of AR under different conditions, such as the conversion of malonyl flavonoids into glycosides during solvent heating extraction (Zheng et al., 2019), as well as the transformation of astragaloside Ⅳ during sterilization and storage (Xu et al., 2021). But are these changes similar to or different from the changes occurring during the roasting process? To our knowledge, some investigations have explored the changes that occur within the AR during the roasting process. Chien, et al. (2022) found that the roasting process increased formononetin content. Astragaloside I, astragaloside IV, isoastragaloside I and astragaloside II were lower in PAR than in RAR (Dai, et al., 2020; Shi et al., 2020). Wu et al. (2021) showed that polysaccharides in PAR were differed in molecular weight, monosaccharide composition, glycosidic bonds, and degree of branching. These results indicated that the roasting process causes the chemical variations. However, only a few have been conducted on the use of metabolomics for comprehensively comparing chemical profiles between RAR and PAR (Liu et al., 2018). The mechanism of chemical composition changes involved in conversion reactions remains unclear.

Thus, a more detailed and extensive analysis of the metabolite profile is needed to better understand the differences between RAR and PAR. In the present study, we analyzed the metabolic fingerprint of AR using RRLC-Q/TOF-MS-based non-targeted metabolomics and identified the differences between the two types *via* a series of chemometric analyses. Meanwhile, phytochemical investigation and high-performance liquid chromatography coupled with photodiode array detection and evaporative light-scattering detection (HPLC–PDA-ELSD) quantitative analysis were applied to verify the reliability of non-targeted metabolomics. The possible chemical transformation was also speculated according to the change in content before and after the roasting process. Quantitative analysis of 15 representative compounds in RAR and PAR were performed. Furthermore, the representative malonyl compounds were used to investigate their chemical conversions under simulated roasting process conditions, and the mechanism of chemical variations and transformations of the AR during roasting was proposed for the first time. The transformations from malonyl compounds to acetyl compounds may be the reason for the greater effect of tonifying in PAR.

## Materials and Methods

### Plant Material, References, and Reagents

The roots of Astragalus *membranaceus* (Fisch.) Bge. var. *mongholicus* (Bge.) Hsiao were collected from NeiMengGu and ShanXi Provinces, two genuine producing areas in China. The voucher specimens were identified by Associate Professor Hui Yan in Nanjing University of Chinese Medicine. The dried roots of six samples were stored in a desiccator. The specific information is shown in Supplementary Material (Supplementary Table S1).

References calycosin-7-glucoside (**2**), calycosin (**24**), formononetin-7*-O-*glucoside (**10**), and formononetin (**50**) were purchased from the National Institute for the Control of Pharmaceutical and Biological Products (Beijing, China). Astrapterocarpan-3*-O-*glycoside (**15**), astraisoflavanglycoside (**16**), astrapterocarpan (**53**), astragaloside I (**59**), and astragaloside IV (**43**) were purchased from Nanjing Jin Yibai Biological Technology Co., Ltd., (Nanjing, China). The purities of these nine reference compounds were greater than 98.0%. The malonyl glycosides calycosin-7*-O-*glycoside-6ʺ*-O-*malonate (**5**), formononetin-7*-O-*glycoside-6ʺ*-O-*malonate (**22**), astrapterocarpan-3*-O-*glycoside-6′*-O-*malonate (**25**), astraisoflavanglycoside-6ʺ*-O-*malonate (**28**), and malonylastragaloside I (**61**) were isolated and identified from AR in our laboratory previously (Zheng et al., 2019). Five acetyl glycosides, calycosin-7*-O-*glycoside-6ʺ*-O-*acetyl (**9**), formononetin-7*-O-*glycoside -6ʺ*-O-*acetyl (**33**), astrapterocarpan-3*-O-*glycoside-6′*-O-*acetyl (**34**), astraisoflavanglycoside -6ʺ*-O-*acetyl (**41**), and acetylastragaloside I (**63**), were isolated from the roasting AR in this study. Their structures were clarified by MS, as well as 1D and 2D NMR analyses. The purities of the isolated compounds were above 97.0%, as determined by HPLC–PAD-ELSD using the peak-area normalization method. In addition, the number in parentheses after the references correspond to those in Table 1.

**TABLE 1:** Data for identification of the metabolites from raw and roasting Astragali Radix by LC-Q/TOF-MS.

No	RT (min)	[M+H]^+^/[M+NH_4_]^+^/[M+Na]^+^	Molecular Formula	Error (ppm)	[Aglycone+H]^+^/[Aglycone+H-H_2_O]^+^	MS^n^ (Characteristic fragment ions)	Identification	Classification	Accumulation
1	12.45	447.1293/469.1112	C_18_H_24_O_3_	1.6	285.0742	270.0527, 253.0507, 225.0544, 213.0563, 197.0566, 137.0248	Isomer calycosin-7*-O-*Glc	Isoflavone	+
					[M+H-Glc]^+^				
2	12.94	447.1286/469.1113	C_22_H_22_O_10_	−1.1	285.0748	270.0507, 253.0484, 225.0535, 213.0539, 197.0594, 137.0234	Calycosin-7*-O-*Glc	Isoflavone *^a^* ^*,*^ *^b^*	+
					[M+H-Glc]^+^				
3	13.97	477.1387/—	C_23_H_24_O_11_	1.9	315.0870	300.0650, 299.0543, 283.0569, 195.0436, 167.0362	Odoratin-7*-O-*Glc	Isoflavone	/
					[M+H-Glc]^+^				
4	17.33	533.1289/555.1123	C_25_H_24_O_13_	−0.1	285.0749	270.0517, 253.0486, 225.0543, 213.0520, 197.0587	Isomer calycosin-7*-O-*Glc-6″*-O-*Mal	Isoflavone	−
					[M+H-Glc-Mal]^+^				
5	18.58	533.1280/555.1121	C_25_H_24_O_13_	−1.8	285.0744	270.0508, 253.0485, 225.0542, 213.0543, 197.0593, 137.0242	Calycosin-7*-O-*Glc-6′*-O-*Mal	Isoflavone *^a^* ^*,*^ *^b^*	−
					[M+H-Glc-Mal]^+^				
6	19.11	449.1431/466.1706/471.1267	C_22_H_24_O_10_	−0.8	287.0923	269.0858, 255.0670, 227.0717, 177.0534, 163.0381,153.0552, 138.0318	10-dihydroxy-9-methoxy-pterocarpan-3*-O-*Glc	Pterocarpan	+
					[M+H-Glc]^+^				
7	19.31	489.1391/511.1211	C_24_H_24_O_11_	−0.1	285.0749	270.0519, 253.0493, 225.0541, 213.0545, 137.0240	Isomer calycosin-7*-O-*Glc-6″*-O-*Ac	Isoflavone	++
					[M+H-Glc-Ac]^+^				
8	21.62	519.1128/541.0948	C_24_H_22_O_13_	−1.0	271.0599	433.1251, 253.0542, 243.0639, 215.0682, 197.0597, 153.0254	3′,4′-dihydroxyisoflavone-7*-O-*Glc-6″*-O-*Mal	Isoflavone	−
					[M+H-Glc-Mal]^+^				
9	22.02	489.1386/	C_24_H_24_O_11_	−0.7	285.0758	270.0508, 253.0483, 225.0537, 213.0536, 197.0591, 137.0235	Calycosin-7*-O-*Glc-6″*-O-*Ac	Isoflavone *^b^*	++
		511.1207			[M+H-Glc-Ac]^+^				
10	22.92	431.1377/453.1154	C_22_H_22_O_9_	1.1	269.0804	254.0576, 237.0543, 226.0622, 213.0906, 197.0600	Formononetin-7*-O-*glucoside	Isoflavone *^a^* ^*,*^ *^b^*	+
					[M+H-Glc]^+^				
11	23.38	461.1434/—	C_23_H_24_O_10_	−1.8	299.0917	284.0670, 256.0739, 243.1007, 239.0683, 211.0724	Cladrin-7*-O-*Glc	Isoflavone *^b^*	+
					[M+H-Glc]^+^				
12	23.48	—/612.2278/617.1829	C_28_H_34_O_14_	−1.4	301.1067	463.1617, 191.0696, 167.0701, 152.0460	Astrapterocarpan-3*-O-*Glc-2′*-O-*xyl	Pterocarpan	/
13	23.82	535.1441/552.1713/557.1269	C_25_H_26_O_13_	−1.0	287.0910	499.1220, 371.0999, 311.0922, 255.0652, 177.0544, 153.0549, 147.0447, 138.0311, 123.0499	10-dihydroxy-9-methoxypterocarpan-3*-O-*Glc-6′*-O-*Mal	Pterocarpan *^b^*	−
					[M+H-Glc-Mal]^+^				
14	23.98	549.1238/571.1054	C_25_H_24_O_14_	−0.2	301.0720	286.0459, 269.0450, 241.0504, 153.0196	Pratensein-7*-O-*Glc-6″*-O-*Mal	Isoflavone *^b^*	−
					[M+H-Glc-Mal]^+^				
15	25.79	463.1584/480.1845/485.1425	C_23_H_26_O_10_	−0.5	301.1080	269.0806, 191.0702, 167.0698, 152.0474, 147.0441, 123.0465	Astrapterocarpan-3*-O-*Glc	Pterocarpan *^a^* ^*,*^ *^b^*	+
					[M+H-Glc]^+^				
16	26.93	465.1751/482.2019/487.1571	C_23_H_28_O_10_	−0.9	303.1220	193.0860, 181.0862, 167.0701, 161.0620, 152.0461, 133.0656, 123.0452	Astraisoflavanglycoside	Isoflavan *^a^* ^*,*^ *^b^*	+
					[M+H-Glc]^+^				
17	27.44	—/698.2292/703.1839	C_31_H_36_O_17_	0.2	301.1066	549.1581, 191.0686, 167.0702, 152.0474	Astrapterocarpan-3*-O-*Glc-6′*-O-*Mal-2′*-O-*xyl	Pterocarpan *^b^*	−
					[M+H-Glc-Xyl-Mal]^+^				
18	27.57	463.1232/485.1047	C_22_H_22_O_11_	−0.6	301.0700	283.0610, 273.0742, 259.0614, 231.0657, 217.0873, 203.0706, 167.0335	Pratensein-7*-O-*glucoside	Isoflavone	/
					[M+H-Glc]^+^				
19	27.78	491.1555/508.1826/513.1374	C_24_H_26_O_11_	−0.3	287.0935	255.0652, 177.0537, 153.0552, 147.0444, 123.0453	10-dihydroxy-9-methoxypterocarpan-3*-O-*Glc-6′*-O-*Ac	Pterocarpan *^b^*	++
					[M+H-Glc-Ac]^+^				
20	27.80	517.1337/539.1166	C_25_H_24_O_12_	−0.7	269.0802	254.0568, 237.0543, 213.0912, 197.592	Isomer formononetin-7*-O-*Glc-6″*-O-*Mal	Isoflavone	−
					[M+H-Glc-Mal]^+^				
21	28.04	547.1440/569.1229	C_26_H_26_O_13_	0.1	299.0916	298.1287, 284.0666, 243.1011, 211.0747, 166.0250, 138.0504, 121.0681	Cladrin-7*-O-*Glc-6″*-O-*Mal	Isoflavone *^b^*	−
					[M+H-Glc-Mal]^+^				
22	28.20	517.1334/539.1154	C_25_H_24_O_12_	−1.3	269.0799	254.0571, 237.0546, 226.0624, 213.0911, 197.0592	Formononetin-7*-O-*Glc-6″*-O-*Mal	Isoflavone *^a^* ^*,*^ *^b^*	−
					[M+H-Glc-Mal]^+^				
23	28.65	515.1541/537.1358	C_26_H_26_O_11_	−1.3	285.0753	270.0517, 253.0493, 225.0540	Calycosin-7*-O-*Rha-2″,3″-di*-O-*Ac	Isoflavone *^b^*	++
					[M+H-Rha-2Acl]^+^				
24	29.07	285.0756/307.0577	C_16_H_12_O_5_	−0.5	—	270.0500, 269.0426, 253.0474, 225.0526, 213.0526, 137.0232	Calycosin	Isoflavone *^a^*	/
25	29.96	549.1603/566.1858/571.1419	C_26_H_28_O_13_	−1.0	301.1052	513.1370, 495.1214, 409.1260, 273.1115, 269.0798, 191.0694, 167.0689, 123.0453	Astrapterocarpan-3*-O-*Glc-6′*-O-*Mal	Pterocarpan *^a^* ^*,*^ *^b^*	−
					[M+H-Glc-Mal]^+^				
26	30.34	549.1596/566.1863/571.1422	C_26_H_28_O_13_	−1.2	301.1071	273.1130, 269.0810, 241.0873, 191.0711, 167.0701, 147.0477	Isomer astrapterocarpan-3*-O-*Glc-6′*-O-*Mal	Pterocarpan	−
					[M+H-Glc-Mal]^+^				
27	30.58	973.5002/995.4810	C_48_H_76_O_20_	−0.1	471.3470	827.4383, 811.4496, 665.3891, 647.3794, 635.4125, 629.3663, 489.3588, 453.3357, 441.3328, 435.3325	Robinioside B	Oleanane	/
					[M+H-GlcA-				
					Glc-Rha]^+^				
28	30.89	551.1757/568.2018/573.1575	C_26_H_30_O_13_	−0.4	303.1227	515.1472, 411.1447, 231.0640, 193.0861, 167.0701, 147.0440, 123.0456	Astraisoflavanglycoside-6″*-O-*Mal	Isoflavan *^a^* ^*,*^ *^b^*	−+
					[M+H-Glcl-Mal]^+^				
29	31.19	827.4429/849.4251	C_42_H_66_O_16_	0.6	471.3474	665.3569, 647.3793, 629.3657, 453.3340, 441.3397, 435.3260	Astraisoolesaponins C1	Oleanane	/
					[M+H-GlcA-Glc]^+^				
30	31.32	551.1754/568.2021/573.1507	C_26_H_30_O_13_	−0.6	303.1230	455.1340, 213.0529, 181.0854, 167.0701, 123.0454	Isome astraisoflavanglycoside-6″*-O-*Mal	Isoflavan	−
					[M+H-Glcl-Mal]^+^				
31	31.43	—/654.2386	C_30_H_36_O_15_	−1.0	301.1065	505.1707, 269.0827, 191.0695, 167.0702, 152.0470	Astrapterocarpan-3*-O-*Glc-6′*-O-*Ac-2′*-O-*xyl	Pterocarpan *^b^*	++
		/703.1839			[M+H-Glc-Xyl-Ac]^+^				
32	31.92	989.5305/1011.5131	C_49_H_80_O_20_	−1.1	473.3632	827.4762, 647.4192, 629.4069, 617.4098, 611.3879, 455.3508, 437.3414, 419.3310, 305.1588, 175.0600, 157.0491, 143.1069	Agroastragalosides Ⅳ	Cycloastragenol	/
					[M+H-2Glc-Xyl-Ac]^+^				
33	32.09	473.1438/	C_24_H_24_O_10_	−1.5	269.0806	455.2738, 254.0582, 237.0527, 213.0904, 198.0618, 163.0319	Formononetin-7*-O-*Glc-6″*-O-*Ac	Isoflavone *^b^*	++
		495.1252			[M+H-Glc-Ac]^+^				
34	32.25	505.1699/	C_25_H_28_O_11_	−1.1	301.1076	269.0818, 241.0863, 191.0700, 167.0707, 152.0469, 147.0450, 123.0460	Astrapterocarpan-3*-O-*Glc-6′*-O-*Ac	Pterocarpan *^b^*	++
		522.1968/			[M+H-Glc-Ac]^+^				
		527.1522							
35	32.35	503.1548/	C_25_H_26_O_11_	−0.4	299.0960	284.0662, 256.0725, 243.1017, 239.0713, 166.0297	Cladrin-7*-O-*Glc-6″*-O-*Ac	Isoflavone *^b^*	++
		525.1370			[M+H-Glc-Ac]^+^				
36	32.63	947.5216/	C_47_H_78_O_19_	0.1	473.3599	785.4616, 653.4421, 635.4156, 605.4029, 473.3599, 455.3506, 437.3399, 419.3198, 297.2209	Astragaloside Ⅴ/Ⅵ/Ⅶ	Cycloastragenol	/
		964.5477			[M+H-2Glc-Xyl-H_2_O]^+^				
37	33.07	507.1861/	C_25_H_30_O_11_	−1.9	303.1220	411.1449, 205.0706, 167.0706, 123.0456	Isomer astraisoflavangly-coside-6″*-O-*Ac	Isoflavan *^b^*	++
		524.2119/			[M+H-Glc-Ac]^+^				
		529.1674							
38	33.20	771.2482/	C_38_H_42_O_17_	−1.7	301.1061	609.1954, 309.0973, 291.0867, 191.0695, 177.0549, 167.0698	Astrapterocarpan-3*-O-*Glc-6′*-O-*coumaroylglucoside	Pterocarpan	/
		788.2749/793.2303			[M+H-2Glc-Cou]^+^				
39	33.58	505.1704/	C_25_H_28_O_11_	−0.5	301.1078	269.0817, 241.0854, 191.0708, 167.0706, 152.04, 147.1417	Pratensein-7*-O-*Glc-6″*-O-*Ac	Isoflavone *^b^*	++
		522.1965/527.1516			[M+H-Glc-Ac]^+^				
40	34.33	827.4784/	C_43_H_70_O_15_	−0.4	473.3648	665.4148, 647.4040, 629.4045, 611.4043, 491.3611, 455.3530, 437.3371, 419.3255, 175.0592, 157.0495, 143.1060	Astragaloside Ⅱ isomer	Cycloastragenol	/
		849.4593			[M+H-Glc-Xyl-Ac-H_2_O]^+^				
		—							
41	34.43	507.1853/	C_25_H_30_O_11_	−0.8	303.1223	471.1643, 411.1442, 393.1331, 231.0655, 193.0861, 181.0863, 167.0698, 165.0551, 147.0447, 133.0653, 123.0455	Astraisoflavanglycoside-	Isoflavan *^b^*	++
		524.2120/529.1673			[M+H-Glc-Ac]^+^		6″*-O-*Ac		
42	34.82	271.0602/—	C_15_H_10_O_5_	0.4	—	270.1998, 253.0533, 243.0651, 153.0185	Genistein	Isoflavone	/
43	34.95	785.4599/	C_41_H_68_O_14_	−2.0	473.3648	665.4148, 647.4040, 629.4045, 611.4043, 455.3530, 437.3371, 419.3255, 175.0592, 157.0495, 143.1060	Astragaloside IV	Cycloastragenol *^a^*	/
		802.4939/807.4486			[M+H-Glc-Xyl-H_2_O]^+^				
44	35.91	827.4787/	C_43_H_70_O_15_	0.4	473.3607	647.4092, 629.3905, 455.3415, 437.3420, 419.3354, 157.0501, 143.1075	Astragaloside Ⅱ	Cycloastragenol	/
		849.4593/			[M+H-Glc-Xyl-Ac-H_2_O]^+^				
45	36.41	1031.5422/	C_44_H_86_O_26_	−5.6	473.3579	869.4663, 815.4291, 689.4361, 671.4125	Agroastragalosides Ⅲ	Cycloastragenol	/
		1053.5209			[M+H-2Glc-Xyl-2Ac-H_2_O]^+^	653.4017, 455.3520, 437.3390, 419.3292, 217.0700, 157.0501, 143.1071			
46	36.58	301.0704/—	C_16_H_12_O_6_	−0.9	—	286.0476, 285.0399, 269.0449, 241.0500, 229.0500, 213.0550, 153.0191	Pratensein	Isoflavone	/
47	36.88	871.4672/	C_44_H_70_O_17_	−1.6	473.3579	853.4390, 835.4369, 709.4331, 691.4061, 673.3876, 655.3879, 491.3705, 455.3507, 437.3408, 419.3302, 143.1080, 125.0984	Astragaloside IV-6*-O-*Glc-4″*-O-*Mal	Cycloastragenol *^b^*	−
		888.4925/			[M+H-Glc-				
		893.4476			Xyl-Mal-H_2_O]^+^				
48	37.75	827.4776/	C_43_H_70_O_15_	−1.4	473.3597	647.4095, 629.4040, 617.4217, 611.3907,	Isoastragalosides Ⅱ	Cycloastragenol	/
		844.5038/			[M+H-Glc-	491.3782, 455.3506, 437.3408, 419.330,			
		849.4595			Xyl-Ac-H_2_O]^+^	175.0602, 157.0500, 143.1074			
49	38.48	943.5248/	C_48_H_78_O_18_	−1.4	459.3816	797.4668, 781.4731, 763.4607, 635.4140, 617.4015, 605.4372, 599.3912, 581.3830, 441.3714, 423.3615, 405.3519	Soyasaponin Ⅰ	Oleanane	/
		965.5061			[M+H-Rha-				
		—			Glc-GlcA]^+^				
50	39.59	269.0806/	C_16_H_12_O_4_	−2.0	—	254.0560, 253.0473, 237.0530, 226.0608, 225.2530, 197.0578, 181.0639, 169.0640	Formononetin	Isoflavone *^a^*	/
51	40.09	797.4667/	C_42_H_68_O_14_	−1.9	459.3885	635.4100, 617.4002, 599.3926, 581.3911, 441.3725, 423.3603, 411.3604, 405.3504	3β*-O-*[*β*-D-GlcA-(1→2)-	Oleanane	/
		819.4489			[M+H-GlcA-Glc]^+^		*β*-D-Glc]-oleanane-12-en-22*β*, 24-diol		
52	40.31	299.0917/	C_17_H_14_O_5_	1.0	—	284.0673, 256.0723, 243.1020, 227.0705, 211.0753, 168.0557	7-hydroxy-3′,4′dimethoxyisoflavone	Isoflavone	/
53	40.95	301.1068/	C_17_H_16_O_5_	−0.8	—	269.0811, 241.0861, 226.0621, 197.0596, 181.0650, 167.0702, 152.0478, 134.0376	Astrapterocarpan	Pterocarpan *^a^*	/
54	41.3	827.4796/	C_43_H_70_O_15_	−0.7	473.3589	809.4734, 665.4156, 647.4289, 629.4009, 611.3928, 455.3515, 437.3400, 419.3312, 175.0609, 143.1073	Astragaloside IV-6*-O-*Glc-4″*-O-*Ac	Cycloastragenol *^b^*	++
		844.5047/			[M+H-Glc-				
		849.4587			Xyl-Ac-H_2_O]^+^				
55	41.49	941.5098/	C_48_H_76_O_18_	−0.7	457.3673	795.4489, 779.4530, 633.3946, 615.3870, 623.4230, 597.3765, 439.3560, 421.3462	3β*-O-*[D-GlcA-(1→2)-	Oleanane	/
		963.4906			[M+H-GlcA-		D-Glc-(1→2)-L-Rha]-oleanane-12-ene-30-oic acid		
		—			Glc-Rha]^+^				
56	41.63	303.1218/	C_17_H_18_O_5_	−3.0	—	193.0867, 167.0709, 161.0606, 152.0476, 133.0661, 123.0455	Isomucronulatol	Isoflavan	/
57	42.73	913.4790/	C_46_H_72_O_18_	−0.2	473.3689	733.4063, 715.4038, 697.3660, 679.3859, 455.3524, 419.3302, 261.0589, 143.1075	Malonylastragaloside Ⅱ	Cycloastragenol *^b^*	−
		930.5031			[M+H-Glc-				
		935.4586			Xyl-Ac-Mal-H_2_O]^+^				
58	43.53	867.4724/	C_45_H_70_O_16_	−1.5	471.3530	849.4644, 687.4105, 669.3979, 453.3353, 435.3259, 417.3156, 217.0705, 157.0505	3β*-O-*[D-glc-(1→2)-D-xyl-	Oleanane	/
		889.4547			[M+H-Glc-		2″,4″-di*-O-*Ac]-oleanane-		
		—			Xyl-2Ac]^+^		12(13)-en-20-glabrolide		
59	43.92	869.4885/	C_45_H_72_O_16_	−0.9	473.3588	851.4777, 833.4667, 689.4271, 671.4119, 653.4033, 455.3526, 437.3409, 419.3303, 297.2208, 217.0709, 157.0505, 143.1079	Astragaloside I	Cycloastragenol *^a^*	/
		886.5150/			[M+H-Glc-				
		891.4693			Xyl-2Ac-H_2_O]^+^				
60	45.58	869.4875/	C_45_H_72_O_16_	−2.1	473.3612	833.4602, 689.4264, 671.4151, 653.4025, 635.3856, 455.3496, 437.3383, 419.3290, 217.0711, 199.0606, 157.0503, 143.1067	Acetylastragaloside Ⅱ	Cycloastragenol *^b^*	++
		886.5138/			[M+H-Glc-				
		891.4697			Xyl-2Ac-H_2_O]^+^				
61	46.54	955.4896/	C_48_H_74_O_19_	−0.1	473.3763	775.4240, 739.4046, 721.3988, 455.3542, 437.3418, 419.3320, 303.0715, 243.0496, 157.0497, 143.1076	Malonylastragaloside I	Cycloastragenol *^a^* ^*,*^ *^b^*	−
		972.5143			[M+H-Glc-				
		977.4697			Xyl-2Ac-Mal-H_2_O]^+^				
62	48.21	869.4883/	C_45_H_72_O_16_	−1.2	473.3626	671.4070, 653.4092, 635.3974, 455.3540, 437.3401, 419.3315, 297.2274, 217.0737, 157.0497, 143.1064, 125.0937	Neoastragalosides Ⅰ	Cycloastragenol	/
		866.5148/			[M+H-Glc-				
		891.4699			Xyl-2Ac-H_2_O]^+^				
63	53.58	911.4985/	C_47_H_74_O_17_	−1.5	473.3626	893.4845, 875.4737, 731.4336, 713.4218, 695.4122, 455.3507, 437.3402, 419.3296, 259.0803, 199.0596, 157.0494, 143.1068, 139.0393, 125.0927, 97.0309	Acetylastragaloside I	Cycloastragenol *^b^*	++
		928.5231/			[M+H-Glc-				
		933.4799			Xyl-3Ac-H_2_O]^+^				

Glc, glycoside; Xyl, xylose; Rha, rhamnoside; GlcA, glucuronide; Cou, coumaroyl; Mal, malonate; Ac, acetyl.; /, the intensity in PAR had no noticeable change than RAR; +, the intensity in PAR was increased, 0.01< *p* < 0.05; ++, the intensity in PAR was significantly increased, *p* < 0.01; −, the intensity in PAR was significantly reduced, *p* < 0.01.

a Identified by reference standards.

b Compounds identified as potential differential metabolites.

LC–MS/MS-grade acetonitrile, methanol, and formic acid were purchased from Merck (Darmstadt, Germany). Distilled water was further purified by a Milli-Q system (Millipore, Milford, MA, United States).

### Sample Preparation

Each batch of AR was cleaned, sliced, and dried at 60°C for 1 h to obtain RAR. PAR sample was prepared with RAR using the roasting method described in the Chinese Pharmacopoeia (Pharmacopoeia of the People’s Republic of China, 2020): RAR (500 g) was roasted on a stove at 150°C until the slices surface was dark yellow. Subsequently, they were taken out, cooled, and weighed. About 477 g of PAR (equivalent to 500 g RAR) was obtained.

The RAR and PAR were pulverized and sifted through a 60-mesh sieve to obtain a homogeneous powder. Each sample was accurately weighed, concentrated to 1.00 g (equivalent to RAR)/mL, and extracted by ultrasonication (500 W power, 40 kHz frequency) with methanol containing 0.1% (v/v) formic acid for 30 min and then cooled at room temperature. The mass was determined, the weight loss was made up for, and the sample solution was shaken well. The sample solution was centrifuged at 12,000 rpm for 10 min. The supernatant was filtered through a 0.22 µm filter, and an aliquot of 5 µl was taken for RRLC-Q/TOF-MS analysis.

A 25 ml solution of the filtrates was evaporated to dryness under vacuum at 40°C. The dry residue was dissolved in a small amount of methanol, transferred into a 5 ml brown volumetric flask, made up to volume with methanol containing 0.1% (v/v) formic acid, and then filtered using a 0.45 µm filter. A 20 µl aliquot was then injected into the HPLC–PDA-ELSD system for quantitative analysis.

### Reference Solution Preparation

Individual reference solutions of 15 compounds for quantitative analysis were prepared in methanol (0.1% formic acid aqueous solution). The appropriate volume of each reference solution was added to a 50 ml volumetric flask and diluted with methanol (contained 0.1% formic acid) to obtain the mixed stock standard solution, in which the concentrations of the analytes were as follows: calycosin-7-glucoside at 106 μg/ml, calycosin-7*-O-*glycoside-6ʺ*-O-*malonate at 198 μg/ml, calycosin-7*-O-*glycoside-6′*-O-*acetyl at 203 μg/ml, calycosin at 33 μg/ml, formononetin-7*-O-*glucoside at 114 μg/ml, formononetin-7*-O-*glycoside-6ʺ*-O-*malonate at 86 μg/ml, formononetin-7*-O-*glycoside-6ʺ*-O-*acetyl at 67 μg/ml, formononetin at 20 μg/ml, astraisoflavanglycoside-6ʺ*-O-*malonate at 210 μg/ml, astraisoflavanglycoside-6ʺ*-O-*acetyl at 203 μg/ml, astrapterocarpan-3*-O-*glycoside-6′*-O-*malonate at 227 μg/ml, astrapterocarpan-3*-O-*glycoside-6′*-O-*acetyl at 200 μg/ml, astragaloside I at 167 μg/ml, malonylastragaloside I at 158 μg/ml, and acetylastragaloside I of 186 μg/ml. Working standard solutions for calibration curves were prepared using a serial dilution method. All of the solutions were stored in a refrigerator at 4°C.

### RRLC-MS/MS Spectrometric Conditions

We used the Eclipse XDB-C_18_ (4.6 mm × 250 mm, 5 μm, Agilent, CA, United States) as the analytical column. The mobile phase consisted of solvent A (acetonitrile) and solvent B (0.1% formic acid in water). The optimized gradient elution was as follows: 0–25 min, 15%–32% A; 25–50 min, 32%–62% A; and 50–60 min, 62%–62% A. All separations were at 25°C and a flow rate of 1.0 ml/min. The injection volume was 5 µl.

The mass spectrometry determination was performed on a quadrupole time-of-flight mass spectrometer (TripleTOF 5600 system, AB Sciex) with an electrospray source in the positive ion mode. The automatic data-dependent information product-ion spectra (IDA-MS/MS) without any predefinition of the ions were recorded within a mass range m/z of 100–1500. The conditions of the ESI source were as follows: nitrogen gas for nebulization at 55 psi, heater gas pressure at 55 psi, curtain gas at 35 psi, temperature of 500°C, and ion spray voltage at 5,500 V in positive ion mode. The acquisition of a survey Q-TOF/MS spectrum was done under high-resolution settings. The optimized declustering potential and collision energy were respectively set at 80 and 15 eV in positive ion mode. A collision energy setting at 35 ± 15 eV was applied for collision-induced dissociation (CID).

### Isolation and Identification of Acetyl Compounds

PAR (5 kg) was exhaustively extracted by refluxing with 30% ethanol (40 L × 2; each extraction lasted 1 h). The extracts were combined and concentrated to about 30 L under vacuum at 60°C. The condensed solution was passed over a microporous resin column (3 L, 30–60 mesh, 8 × 120 cm) at a flow rate of 100 ml/min and then eluted with H_2_O (6 L, 100 ml/min flow rate), EtOH–H_2_O (20:80, 6 L, 100 ml/min flow rate), and EtOH–H_2_O (80:20, 12 L, 100 ml/min flow rate). The EtOH–H_2_O (80:20) solution was concentrated *in vacuo* at 60°C. The residue (a total of about 150 g) was then separated by silica gel column chromatography (2000 g, 200–300 mesh) to obtain fractions (Frs.) 1–50 using a gradient elution of EtOAc–MeOH (100:0–90:10, v/v). All fractions were analyzed using an HPLC–PDA-ELSD system. Selected similar composition fractions were pooled and concentrated. White amorphous powder was precipitated from Frs. 10–13 and Frs. 28–30, respectively, and recrystallized to obtain **C-1** (165 mg) and **C-5** (89 mg). In addition, Frs. 3 and 4 was subjected to HPLC on a semi-preparative C_18_ column (9.4 mm × 250 mm, 5 μm, Agilent, CA, United States) using CH_3_CN–H_2_O–HCOOH (35:65:0.1, v/v/v) as the eluent to give **C-3** (65 mg) and **C-4** (53 mg). The same separation procedure was used to isolate **C-2** (73 mg) from Frs. 7 and 8.

The purified compounds were characterized by LC–MS and NMR analyses. The MS spectra were recorded on an AB Sciex Triple TOFTM 5600 mass spectrometer instrument (AB Sciex, Los Angeles, CA, United States) in positive ion mode. ^1^H-NMR and ^13^C-NMR spectra were recorded with an ASR-500 NMR spectrometer (Bruker, Fällanden, Switzerland). TMS was used as an internal standard, and the specimens were dissolved in DMSO-d_6_ (dimethylsulfoxide).

### HPLC-PDA-ELSD Analysis for Quantitation

Quantitative analyses were performed on a Waters Series 2695 liquid chromatograph (Waters Technologies, Milford, MA, United States) consisting of a dual pump, an autosampler, a PDA coupled with an ELSD (Alltech Associates, Deerfield, United States), and Eclipse XDB-C_18_ analytical column (4.6 mm × 250 mm, 5 μm, Agilent, CA, United States). The mobile phase consisted of (A) acetonitrile and (B) distilled water containing 0.1% (v/v) formic acid using a gradient elution: linear from 15 to 32% B (0–25 min), linear from 32 to 62% B (25–50 min), and linear from 62 to 62% B (50–60 min). The column temperature was set at 25°C, and the mobile flow rate was kept at 1.0 ml/min. The injection volume was 20 μl. The PDA chromatographic profile was recorded at 280 nm. The drift tube temperature for ELSD was set at 105°C, and the nebulizing gas flow rate was set at 2.7 L/min.

The stock solution was diluted to provide different concentration ranges. The calibration curve for each compound was plotted with at least six appropriate concentrations in triplicate. For the 12 isoflavonoids recorded by UV detection, the regression equations were calculated in the form of Y = bX + a, while for the three saponins recorded by ELSD detection, the regression equations could be described as ln Y = b ln X + a, where Y and X are peak area and concentration, respectively. The dilute stock solution of the 15 reference compounds was further diluted with methanol containing 0.1% (v/v) formic acid to give a series of concentrations for determining the limits of detection (LOD) and quantification (LOQ). The LOD and LOQ under the present chromatographic conditions were determined at a signal-to-noise (S/N) ratio of 3 and 10, respectively.

The same batch (no. 190901) of RAR and PAR at a ratio of 1:1 was mixed and powdered to obtain the sample for method validation. The precision of the developed assay was determined under optimal conditions by means of six replicate determinations of a mixed standard solution. The repeatability of the method was examined by performing six replicate determinations. The stability was tested at room temperature and analyzed at 0, 2, 4, 6, 8, 10, and 12 h. The relative standard deviation (RSD) values for peak area of each compound were calculated.

The recoveries of the 15 references were determined by adding accurately known amounts of them to approximately 1.0 g of the mixed sample and then performing extraction and analysis were performed as described in Section 2.2. The average recoveries were calculated by the following equation: recovery (%) = (amount found – original amount)/amount spiked × 100%, and RSD (%) = (SD/mean) × 100%.

### Data Processing, Statistical Analysis, and Identification of Metabolites

The LC–Q/TOF-MS raw data of the RAR and PAR samples were initially analyzed using the molecular feature extraction algorithm of the MarkerView software (AB Sciex, Foster City, CA, United States). According to the previous literature (Peralbo-Molina et al., 2012), the molecular feature extraction algorithm took into account all ions exceeding 1,000 counts with a charge state equal to one. The intensity of each ion was normalized and filtered to the total ion count in order to generate a data matrix having an m/z value, R_t_, and normalized peak area. The data matrix from different samples was aligned using a mass accuracy of ± 10 ppm, a retention time range of 5–60 min, retention time tolerance of ± 0.20 min, intensity threshold of 5,000 counts, and exclusion of isotopic peaks. Considering six batches each for RAR and PAR, we set the minimum number of peak appearances was set to six, ensuring that the new components from the PAR would not be lost during the screening process. Importantly, the MarkerView software automatically set the peak value at the new compound position in the RAR to 0, allowing direct comparability between the two kinds of data sets.

The processed data were then imported into SIMCA13.0 (Umetrics, Umea, Sweden) for multivariate analysis using the method described previously (Mais et al., 2017). After dataset pretreatment using mean-centered and Pareto (Par)-scaled mathematical methods, principal component analysis (PCA) and orthogonal partial-least-squares-discriminant analysis (OPLS-DA) multivariate statistical analysis were carried out to investigate the differential metabolites between RAR and PAR. Evaluated models were calculated for over-fitting with methods of the Hotelling’s T2 range and permutation tests. T2 Crit (95%) and T2 Crit (99%) were used to evaluate the reliability of the PCA. R2X and R2Y values were used to describe the performance of the OPLS-DA model prediction performance. S plots were created to find those having the highest discrimination potential between RAR and PAR by visualizing the covariance and correlation within the OPLS-DA data. Metabolic variables with high influence and variable importance in projection (VIP) values of >1.5 was selected for further analysis. Additional criterion for the inclusion of metabolites was that the fold change between the groups compared should be greater than 1.5 (i.e., F.D. 1.5) and *p* < 0.01.

Metabolites that met these criteria were taken as the differential compounds. Their structures were identified on the basis of their mass spectral data using the Metlin database and relevant published literature, and then were confirmed by reference compounds or their fragmentation patterns. Heatmaps of differential metabolites of the two types of AR samples were obtained using Origin Pro 2017 software (OriginLab, MA, United States). The peak areas of the differential metabolites from RAR and PAR were normalized using their median and the data transformed by logarithm and then imported into OriginPro software to generate a heatmap by adjustment of the color bands.

Significant differences were analyzed by using a paired sample *t*-test in GraphPad Prism 7.0 (La Jolla, CA, United States). For all analyses, *p* < 0.01 and *p* < 0.001 were considered statistically significant.

### Chemical Conversion of Malonyl Compounds

The chemical conversion experiment of five malonyl compounds under simulated roasting process condition was as follows. An appropriate amount of calycosin-7*-O-*glycoside-6ʺ*-O-*malonate, formononetin-7*-O-*glycoside-6ʺ*-O-*malonate, astrapterocarpan-3*-O-*glycoside-6′*-O-* malonate, astraisoflavanglycoside-6ʺ*-O-* malonate, and malonylastragaloside I was weighted, and 2 ml of methanol was added. Subsequently, 4 g of silica gel was added to the solution, and the mixture was mixed well. Next, each group of samples was further separated into four portions (1.0 g each portion) and roasted at 150 ± 10°C to study the extent of chemical transformations in the roasting process at different collection points. The roasting durations were 0, 10, 20, and 30 min. After roasting, 0.5 g of each sample was weighed and extracted with 5 ml of methanol in an ultrasonic bath for 30 min at room temperature. The extract was then filtered with 0.45 μm filter for further HPLC–PDA-ELSD analysis.

The HPLC analytical conditions were the same as in section 2.6 except for slight modification of the mobile phase. For efficiency, the mobile phases were optimized as (A) acetonitrile and (B) distilled water containing 0.1% (v/v) formic acid. Analysis was linear from 20 to 60% A (0–20 min) for isoflavonoid analysis and linear from 50 to 70% A (0–20 min) for astragaloside analysis.

## Results

### Structural Characterization by RRLC-QTOF/MS Analysis

For a more comprehensive analysis of chemical constituents in RAR and PAR, the negative- and positive-ion-mode tests were implemented for RRLC-QTOF/MS analysis. The results show that the positive-ion-mode MS/MS spectra of the protonated forms of isoflavonoids and triterpene saponins were more intense and informative than were the negative-ion-mode MS/MS spectra, despite the excellent ionization in negative mode of the parent species, which is consistent with previous reports (Chu et al., 2010; Song et al., 2007).

**FIGURE 1:**
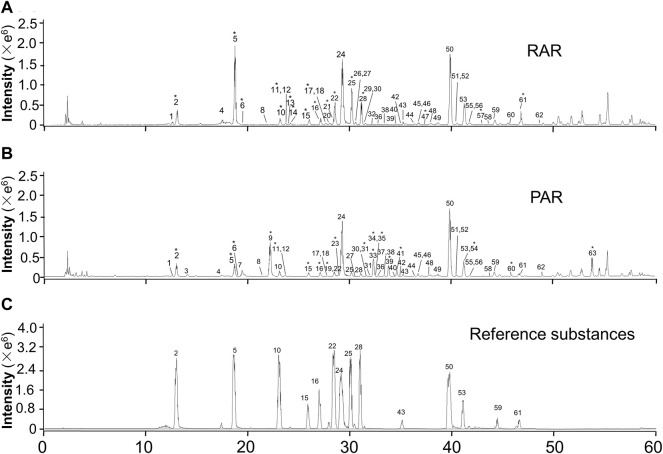
Representative RRLC-Q/TOF-MS (+) total ion chromatograms of RAR **(A)**, PAR **(B)**, and reference substances **(C)**. *Compounds were identified as differential metabolites.

The total ion chromatograms in positive ion modes of RAR and PAR are presented in Figure 1. The reference compounds were initially analyzed to obtain the retention time and characteristic fragmentation pathway data prior to dissecting the samples. Then, characteristic compounds of RAR and PAR were identified by comparing the retention time and mass spectra data with those of the references and the literature data. Fragment data from the MS/MS spectra were used to further confirm the structures of the compounds. A total of 63 metabolites composed of 42 isoflavonoids (23 isoflavones, 13 pterocarpans, and six isoflavans) and 21 triterpene saponins (15 cycloastragenols and six oleananes) were identified in both of the extracts of RAR and PAR. Data obtained from the ESI-MS^n^ analysis on the metabolites are summarized in Table 1. Additionally, taking references as the control, we used the retention time of the 14 components in the RAR and PAR were used for the method validation. The results showed that the RSD of retention time was ≤ 0.3%, demonstrating good reliability of the metabolomics method to a certain degree (Supplementary Table S2).

#### Identification of Isoflavonoids (Isoflavones, Pterocarpans, and Isoflavans)

Isoflavones are one of the most important isoflavonoid ingredients in AR (Song et al., 2007). Compounds 2, 5, 10, 22, 24, and 50 were unambiguously identified as calycosin-7*-O-*glycoside, calycosin-7*-O-*glycoside- 6ʺ*-O-*malonate, formononetin-7*-O-*glucoside, formononetin-7*-O-* glycoside-6ʺ*-O-*malonate, calycosin, and formononetin, respectively, on the basis of R_t_ values and MS spectra of available references. Identification of genistein (**42**), pratensein (**46**), and 7-hydroxy-3′,4′- dimethoxyisoflavone (**52**) was based on their accurate mass values, fragmentation patterns, and related literature (Zhang et al., 2007). The MS^n^ fragmentations of compounds **1**, **3**, **11**, and **18** were dominated by the elimination of glucosyl residue, which gave [aglycone+H]^+^ ions as the base peak. Product ions with low m/z values were the same as those obtained from their aglycones. Compounds **4**, **7–9**, **14**, **20**, **21**, **33**, and **35** were 42 and 86 Da greater than the corresponding isoflavone glycosides and had similar fragmentation ions at the m/z values, implying an additional acetoxyl and malonyl in the structure. Compound **9** produced [M+NH_4_]^+^ and [M+H]^+^ at m/z 511.1207 and 489.1386, respectively. The typical loss of 204 Da (m/z 489.1386, 285.0758) corresponding to the acetylglucoside residue was observed in the MS^2^ spectrum. The other product ions at m/z 270.0508, 253.0483, 225.0537, 213.0536, 197.0591, and 137.0235 were produced from aglycone ion at m/z 285.0758 due to C-ring RDA cleavage, as well as arrangement and successive loss of CH_3_ (15 Da), CO (28 Da), and CH_3_OH (32 Da). These characteristic ions were identical to the MS^2^ spectra of calycosin (**24**), leading to the identification of **9** as calycosin-7*-O-*glycoside-6ʺ*-O-*acetyl. The mass spectra and possible fragmentation pathway are proposed in Supplementary Material (Supplementary Figure S1).

Pterocarpan is another major type of isoflavonoid in AR. Among them, **15**, **25**, and **53** were unambiguously determined as astrapterocarpan-3*-O-*glycoside, astrapterocarpan-3*-O-* glycoside-6′*-O-*malonate, and 3-hydroxy-9,10-dimethoxypterocarpan, respectively, which were confirmed by comparing the retention times and mass spectra with those of the reference standards. Compounds **12**, **17**, **26**, **31**, **34**, **38**, and **39** were derivatives of astrapterocarpan, and the serial characteristic ions (m/z 301.1, 191.1, and 167.1) were specific in their MS^2^ spectra. The successive loss of fragments of 132 and 162 Da/204 Da/248 Da from precursor ions [M+NH_4_]^+^ of **12**, **17**, and **31** offered evidence for the assignment of *-O-*glucoside-2′*-O-*xylosyl, *-O-*glucoside-6′*-O-*malonate-2′*-O-*xylosyl, and *-O-*glucoside-6′*-O-*acetyl-2′*-O-*xylosyl, respectively. Compound **34** was identified as astrapterocarpan-3 *-O-* glycoside-6 ′ *-O-* acetyl. It gave [ M+H] ^+^ and [ M+NH_4_ ] ^+^ at m/z 505.1699 and m/z 522.1968. The MS ^2^ spectrum shows aglycone ion at m/z 301.1076 due to the loss of an acetylglycoside. The product ions at m/z 269.0818 and 241.0863 were derived from the aglycone ion by concurrent loss of CH_3_ OH (32 Da) and CO (28 Da). The ion at m/z 123.0460 was produced from the RDA cleavage of the aglycone ion. In addition, the other product ions were at m/z 191.0700, 167.0707, 152.0469, and 147.0450 because of the losses of the B-ring and C-ring arrangements. The mass spectra and possible fragmentation mechanism are depicted in Supplementary Material (Supplementary Figure S2). In the same manner, other pterocarpan compounds were presumed on the basis of similar cleavage patterns.

Compounds **16**, **28**, **30**, **37**, **41**, and **56** were assigned as isoflavans due to the typical aglycone ion at m/z 303.1220 (isomucronulatol) derived from the characteristic losses of glucose (162 Da), acetylglucoside (204 Da), or malonylglucoside (248 Da). Of these, **16** and **28** were determined as astraisoflavanglycoside and astraisoflavanglycoside-6ʺ*-O-*malonate, respectively, with the reference compounds. Peak **41** produced precursor ions at m/z 507.1853 [M+H]^+^ and m/z 524.2120 [M + NH_4_]^+^, indicating the molecular formula of C_25_H_30_O_11_. The MS^2^ spectra yielded the aglycone ion at m/z 303.1223 by the loss of 204 Da (acetylglucoside), as well as other characteristic ions from aglycone at m/z 193.0861, 181.0863, 167.0698, 165.0551, 147.0447, 133.0653, and 123.0455, identical to the MS^2^ spectra of peak **16**. This led to the identification of **41** as astraisoflavanglycoside-6ʺ*-O-*acetyl Supplementary Material (Supplementary Figure S3). Considering that OH and OCH_3_ can be located at different positions, we presumed compounds **30** and **37** to be isomers of astraisoflavanglycoside-6ʺ*-O-*malonate and astraisoflavanglycoside-6ʺ*-O-*acetyl, respectively.

#### Identification of Triterpene Saponins (Cycloastragenols and Oleananes)

Cycloastragenol-type triterpene saponins, named astragalosides, are major active ingredients in AR. For the same 9,19-cyclolanostane aglycone possessed, astragalosides displayed characteristic ions, including the aglycone residues at m/z 473.3726, 455.3507, 437.3402, 419.3296, as well as 25-hydroxy and 20,24-epoxy residues at m/z 143.1068, 125.0927 (Chu et al., 2010). Neutral losses of sugar moieties such as glucose (162 or 180 Da) and xylose (132 Da) were commonly observed in the MS spectra for prediction of the sugar number and sequences. Other typical losses included C_7_H_10_O_5_ (174 Da), C_9_H_12_O_6_ (216 Da), C_11_H_14_O_7_ (258 Da), and C_12_H_14_O_9_ (302 Da) from [M+H]^+^, which respectively correspond to the presence of one acetyl residue, two acetyl residues, three acetyl residues, and two acetyl and one malonyl residues linked to the xylosyl moiety.

In the present study, compounds **43**, **59**, and **61** were identified undoubtedly on the basis of retention time and mass spectra of available standards. Peak **63** was a cycloastragenol triterpene saponin, which was significantly increased in PAR compared with RAR. The TOF-MS data show that the molecular ion of **63** was at m/z 911.4985 ( [ M+H] ^+^ ), which was calculated as a molecular formula of C_47_ H_74_ O_17_ . When targeted with [ M+H] ^+^ in the MS ^2^ spectrum, fragment ions at m/z 893.4845 and 875.4737 were readily found because of the successive loss of 2 × H_2_ O ascribed to the dihydroxy groups at C-18 and C-25 . The presence of m/z values at 731.4336, 713.4218, 695.4122, and 677.4003 could be assigned to the fragments of [ M+H-Glc-H_2_ O] ^+^ , [ M+H-Glc-2H_2_ O] ^+^ , [ M+H-Glc-3H_2_ O]+, and [ M+H-Glc-4H_2_ O]+, respectively; these indicate that the sugar moiety connection at C-6 was more readily cleaved than that at the C-3-position. The presence of a typical ion at m/z 731.4336 and 473.3626 could be assigned to the fragments of [ M+H- (Glc+H_2_ O)] ^+^ and [ M+H-(Glc+H_2_ O)-(xyl+ 3 Ac)] ^+^ , respectively. These indicate that the glucosyl moiety at the C-6-position was more readily cleaved than the xylosyl moiety at C-3 position. The aglycone ion at m/z 473.3626 further produced the fragment ions at m/z 455.3507, 437.3402, and 419.3296 by successive losses of several water molecules. The characteristic ion at m/z 143, 125 with high intensity was attributed to a 25-hydroxy-20,24-epoxy residue by the cleavage of the bond between C-17 and C-20 and further loss of one water molecule ( Huang et al., 2009 ). It is noteworthy that the ions at m/z 259.0803, 199.0596, 157.0494, and 139.0393, which were assigned to [ xyl+3Ac-H_2_ O] , [ xyl+2Ac-2H_2_ O] , [ xyl+Ac-2H_2_ O] , [ xyl+Ac-3H_2_ O], respectively, also had a relatively high intensity. The possible fragmentation pathway (Huang et al., 2009) of **63** is presented in Supplementary Material (Supplementary Figure S4). The other cycloastragenol-type saponins were tentatively characterized on the basis of the protonated molecular ion, R_t_ values, fragmentation products, and typical neural losses.

Oleananes are another type of triterpene saponins in AR. In addition to the characteristic oleanane of aglycone, the presence of glucose, rhamnose, xylose, and glucuronic acid is the most prominent structural feature in the sugar chain. Considering the fragment pathway of oleanane-type saponins (Zheng et al., 2010), we assigned compounds **27**, **29**, **49**, **51**, **55**, and **58** by comparing their mass spectra with those reported previously.

### Metabolic Profiling Analysis of RAR and PAR

**FIGURE 2:**
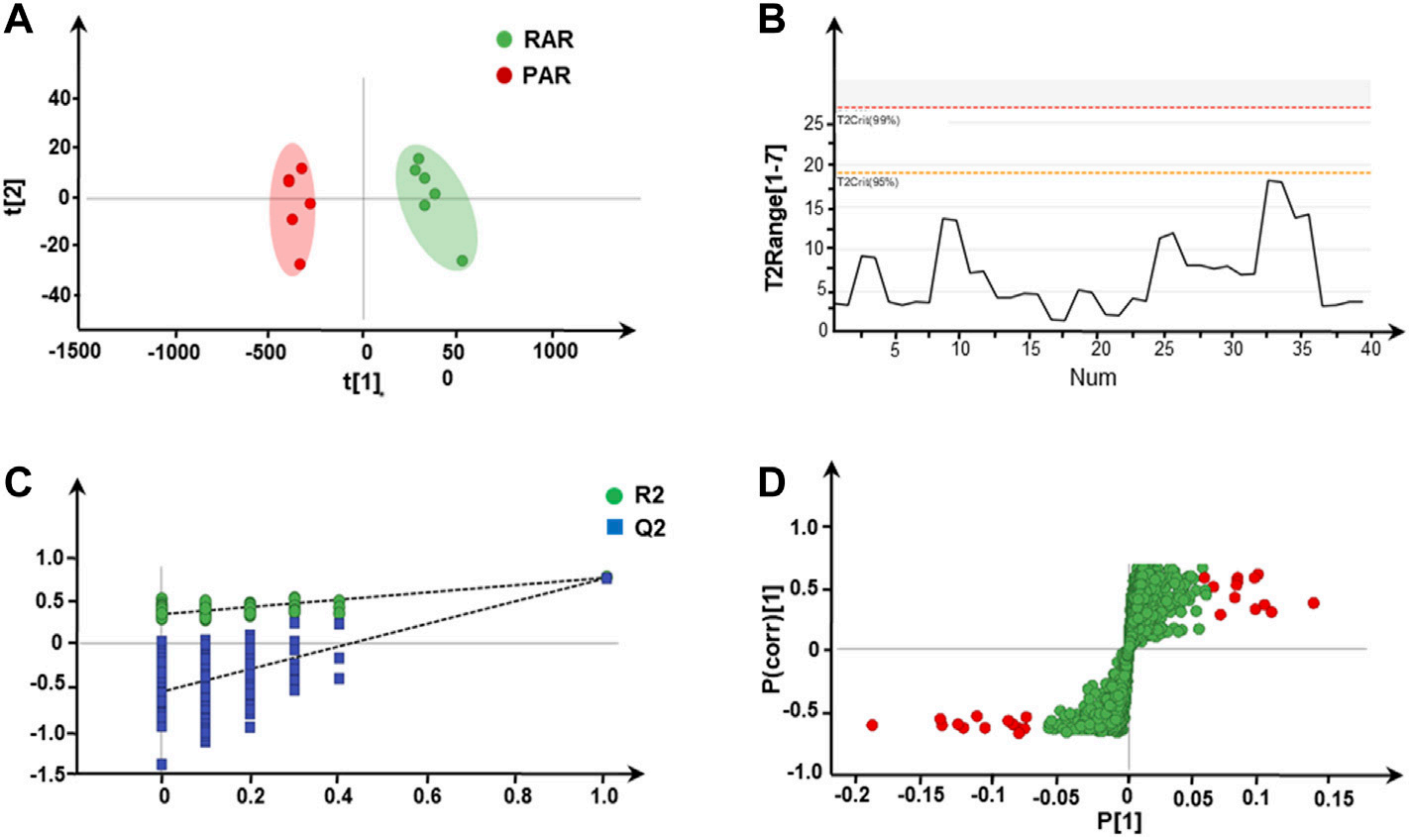
Screening and determination of differential metabolites from two types of AR. **(A)** Unsupervised PCA score plot of RAR and PAR, **(B)** Hotelling’s T2 range line plot, **(C)** presentation of chance permutation at 200 times used for the discrimination between RAR and PAR, and **(D)** S-plot with OPLS-DA analysis of RAR and PAR.

Under the previously optimized and determined positive-ion mode, a total of 1,742 features were extracted from all batches of AR. After normalization and filtering (Zheng et al., 2020), 310 ions were extracted for the PCA. The outcome of unsupervised PCA on 12 batches of AR in positive-ion mode is shown in Figure 2A. The scoring plots of RAR and PAR were divided into two significant clusters, which can be well distinguished from each other; RAR with green cluster is on the right of the central axis, while PAR with red cluster is on the left. In addition, T2Crit (95%) is higher than the T2 range for all samples, suggesting that the PCA model is credible (Figure 2B).

To validate the model of OPLS-DA, a permutation test (*n* = 200) (Figure 2C) was conducted. According to OPLS-DA clustering analysis, the cumulative interpretation parameter R2Y of the established model is 0.994, and the prediction capability parameter Q2 is 0.986, indicating that the model has good identification and prediction capability. The corresponding S-plot OPLS-DA (Figure 2D) displays the ions conducive to distinguishing groups of RAR and PAR. By definition, ions near the origin show little contribution to the separation of groups, whereas those situated farthest from the origin are the most important variables. In Figure 2D, a large number of variables are located near the observed values of the samples, and some components significantly increase (the point at the upper right of the figure), while others obviously decrease (the point at the lower left of the figure), indicating that the PAR products have undergone a significant change in the roasting process.

**FIGURE 3:**
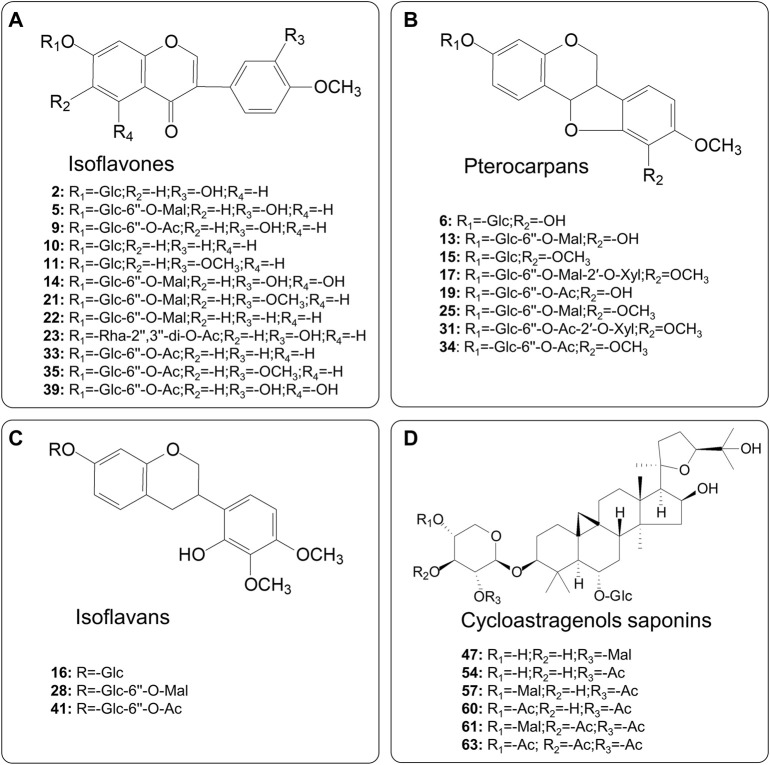
Chemical structures of the identified differential metabolites between RAR and PAR.

After being characterized by multivariate statistical analysis with VIP > 1.5 and *p* < 0.01, 56 variables were selected for visualization and screened for potential different components. The red points in the plot represent the differentially varied components during processing. Overall, 29 differential metabolites composed of 12 isoflavones, eight pterocarpans, three isoflavans, and six cycloastragenol type saponins were characterized and identified on the basis of screening criteria. Their structures are presented in Figure 3.

### Heatmap and Histogram Visualization of Differential Metabolites Between RAR and PAR

**FIGURE 4:**
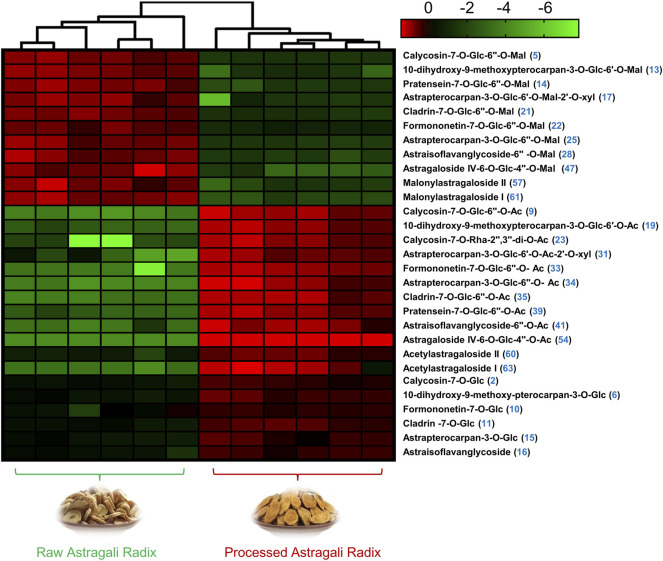
Heatmap visualization from metabolomic analysis indicated that 29 compounds showed different trends of variation in AR before and after roasting.

In order to better visualize metabolite differences between the two forms of AR, the peak areas of 29 differential metabolites were log transformed to generate a heatmap (Figure 4), in which the brighter the colors (or green) and higher (or lower) peak area represent higher (or lower) content in AR. It shows that the RAR and PAR were clearly distinguished on the basis of the clustering relationships of the differential metabolites. Among the 29 differential metabolites, malonyl components, including eight malonyl isoflavonoid glycosides (**5**, **13**, **14**, **17**, **21**, **22**, **25**, and **28**) and three malonyl cycloastragenols (**47**, **57**, and **61**), showed relatively higher levels of enrichment in PAR than those in RAR. Meanwhile, the content of acetyl isoflavonoid glycosides (**9**, **19**, **23**, **31**, **33–35**, **39**, and **41**), isoflavonoid glycosides (**2**, **6**, **10**, **11**, **15**, and **16**), and acetyl cycloastragenols (**54**, **60**, and **63**) in RAR was significantly lower than that of PAR.

Of note, while most malonyl isoflavonoid glycosides decreased, the corresponding acetyl isoflavonoid glycosides and isoflavonoid glycosides in the same structural skeleton increased during the processing. A similar phenomenon has also been observed among the screened differential cycloastragenols. From a structural point of view, the stability of malonyl components is poor, suggesting that the malonyl glycosides may be converted into the corresponding acetyl glycosides and/or glycosides during the roasting process.

### Acetyl Compound Separation and Structural Elucidation

To confirm the structures of differential acetyl compounds characterized by Q-TOF/MS, an efficient isolation protocol using microporous-resin adsorbent chromatography combined with silica column chromatography and preparative high-performance liquid chromatography (pHPLC) was performed. This allowed us to obtain five representative acetyl compounds from PAR. The ^13^C-NMR data of the five compounds are shown in the Supplementary Material (Supplementary Table S3).

**C-1** obtained was a light-yellow powder. Its molecular formula was given as C_24_H_24_O_11_ on the basis of HR-ESI-MS at m/z 489.1385 [M+H]^+^ (calcd 489.1391). The 1H-NMR spectrum shows an ABX spin system with signals at *δ*H 8.05 (1H, d, J = 8.8 Hz) and *δ*H 7.14 (1H, dd, J = 8.8 Hz, J = 2.3 Hz); a signal peak at *δ*H 8.39 (1H, s); and a spin system with signals at *δ*H 7.01 (1H, brs), *δ*H 6.97 (1H, m), and *δ*H 7.07 (1H, d, J = 2.2 Hz), implying that C-1 was a typical isoflavonoid compound. In addition, an anomeric proton at *δ*H 5.17 (1H, d, J = 7.3 Hz), which was correlated with *δ*C 99.7 in the HSQC spectrum, represented the structure containing one sugar moiety. In the MS^n^ spectrum, the typical ion m/z 285.0741, derived from protonated ion m/z 489.1385 [M+H]^+^ by neural loss of C_8_H_12_O_6_ (204 Da), indicated the existence of an acetylglucoside residue in C-1. This result was further confirmed by the signals of one unsaturated quaternary carbon at *δ*C 170.2, one methyl carbon at *δ*C 20.7, one anomeric carbon at *δ*C 99.7, and four tertiary carbons at *δ*C 63.3–76.2. The determination of the linkage sites was verified from the HMBC correlations between *δ*H 5.17 (H-1′) and *δ*C 161.2 (C-7), as well as *δ*H 4.34 (H-6′) and *δ*C 170.2 (C-1ʺ). From these spectroscopic data, the compound **C-1** was deduced to be calycosin-7*-O-*β-D-glycoside-6ʺ*-O-*acetyl (**9**). Correspondingly, **C-2**, **C-3**, and **C-4** were identified as formononetin-7*-O-*glycoside-6ʺ*-O-*acetyl (**33**), astrapterocarpan-3*-O-*glycoside-6′*-O-*acetyl (**34**), and astraisoflavanglycoside-6ʺ*-O-*acetyl (**41**) by a combination of MS/MS and NMR by comparison with previous work (Zhang et al., 2011; Zheng et al., 2019). Their structures and the key HMBC correlations are illustrated in Supplementary Material (Supplementary Figure S5).

**C-5**, a white powder, generated a [M+Na]^+^ ion at m/z 933.4798 and a [M+H]^+^ at m/z 911.4951, which is in agreement with the molecular formula of C_47_H_74_O_17_. Two fragment ion peaks at m/z 731.4336 [M-C_6_H_12_O_6_+H]^+^ and m/z 473.3626 [M-C_6_H_12_O_6_–C_11_H_14_O_7_+H]^+^ suggest the presence of two sugar moieties, which was confirmed with two anomeric protons at *δ*H 4.67 (1H, d, J = 7.6 Hz) and *δ*H 4.15 (1H, d, J = 7.7 Hz), as well as two anomeric carbons at *δ*C 102.3 and *δ*C 103.7. The coupling constants of the anomeric protons indicate that the glycosidic bonds had a β configuration. In addition, the ^13^C-NMR spectrum of **C-5** exhibited 30 carbon resonances assigned to the aglycone moiety consisting of seven methyls, nine methines, seven methylenes (of which four were oxygenated), and seven quaternary carbons (including two oxygenated carbons), implying that **C-5** was a cycloastragenol-type saponin. The determination of the sugar-linkage sites was obtained from the HMBC correlations between *δ*H 4.67 (H-1′) and *δ*C 88.4 (C-3) and between *δ*H 4.15 (H-1ʺ) and *δ*C 78.3 (C-6). Of note, three typical acetyl moieties with three carbonyl carbons at *δ*C 168.8, *δ*C 169.7, and *δ*C 169.7; three methyl carbons at *δ*C 20.6, *δ*C 20.6, and *δ*C 20.5; and the three corresponding methyl protons at *δ*H 1.96 (3H, s), *δ*H 1.96 (3H, s), and *δ*H 1.95(3H, s) are present in the ^13^C-NMR and ^1^H-NMR spectra. The HMBC correlations between the oxygenated proton at *δ*H 4.81 (H-4′) and carbonyl carbon at *δ*C 168.8 (4′-Oac), *δ*H 4.76 (H-3′), and *δ*C 169.7 (3′-Oac), *δ*H 5.18 (H-2′), and *δ*C 169.7 (2′-Oac) identified three acetyl moieties that were linked to the xylosyl group. These were clearly verified by the secondary dissociation mass fragment ions at m/z 259.0805 [xyl+3Ac-H_2_O]^+^, m/z 199.0598 [xyl+2Ac-2H_2_O]^+^, m/z 157.0499 [xyl+Ac-2H_2_O]^+^, and m/z 139.0400 [xyl+Ac-3H_2_O]^+^. Comparing the data with the literature (Chu et al., 2010), we identified compound **C-5** as acetylastragaloside I (**63**). The structure of **C-5** is presented in Supplementary Material (Supplementary Figure S5).

### Quantitative Analysis of the 15 Representative Ingredients by HPLC–PDA-ELSD

To better support the results of non-targeted metabolomics, the 15 representative compounds, which were composed of three non-differential compounds (calycosin, formononetin, and astragaloside I) and 12 differential metabolites (calycosin-7*-O-*glucoside, calycosin-7*-O-*glucoside-6ʺ*-O-* malonate, calycosin-7*-O-*glucoside-6ʺ*-O-*acetyl, formononetin-7*-O-*glucoside, formononetin-7*-O-*glucoside- 6ʺ*-O-*malonate, formononetin-7*-O-*glucoside-6ʺ*-O-*acetyl, astrapterocarpan-3*-O-*glucoside-6ʺ*-O-*malonate, astrapterocarpan-3*-O-*glucoside-6ʺ*-O-*acetyl, astraisoflavanglycoside-6ʺ*-O-*malonate, astraisoflavanglycoside-6ʺ*-O-*acetyl, malonylastragaloside I, and acetylastragaloside I), were used to investigate the content variations of RAR and PAR.

It is well known that a UV detector is very convenient and sensitive for the determination of isoflavonoids. However, compounds such as cycloastragenol-type saponins with very few chromophore groups have poor UV absorption and are therefore difficult to be detected using this type of detector. ELSD is an alternative detector that has been increasingly used as an efficient tool for determining the non-chromophoric compounds in TCMs (Chen et al., 2015). Referring to the previous literatures (Liu et al., 2014), we developed a simple and reliable method of HPLC–PDA-ELSD for analyzing the RAR and PAR.

#### Method Validation

Considering the large variability in content of differential metabolites between RAR and PAR, we mixed RAR and PAR powder of the same batch (no. 190901) at a ratio of 1:1 as the sample for method validation. PDA, and ELSD conditions, linear regression equations, linearity range, correlation coefficients, limit of detection (LOD), and limit of quantitation (LOQ) for the listed 15 compounds under optimized chromatography are shown in Supplementary Material (Supplementary Table S4). The regression equations for isoflavonoid compounds were calculated in the form of Y = bX + a, while the regression equations for the three saponins (**59**, **61**, and **63**) determined by ELSD were described as ln Y = b ln X + a, where Y and X are peak area and concentration, respectively.

As shown in Supplementary Material (Supplementary Table S5), the RSDs of precision of 15 reference compounds were 0.92–2.10%, indicating that the precision of this method was acceptable. The RSDs of repeatability and stability of the 15 analytes were less than 5%, indicating that the 15 compounds were generally stable over 12 h. The percentage of average recoveries ranged from 92.1 to 106.2%, with RSD less than 6.50%, demonstrating that the method was accurate and feasible.

#### Determination of 15 Representative Analytes

**FIGURE 5:**
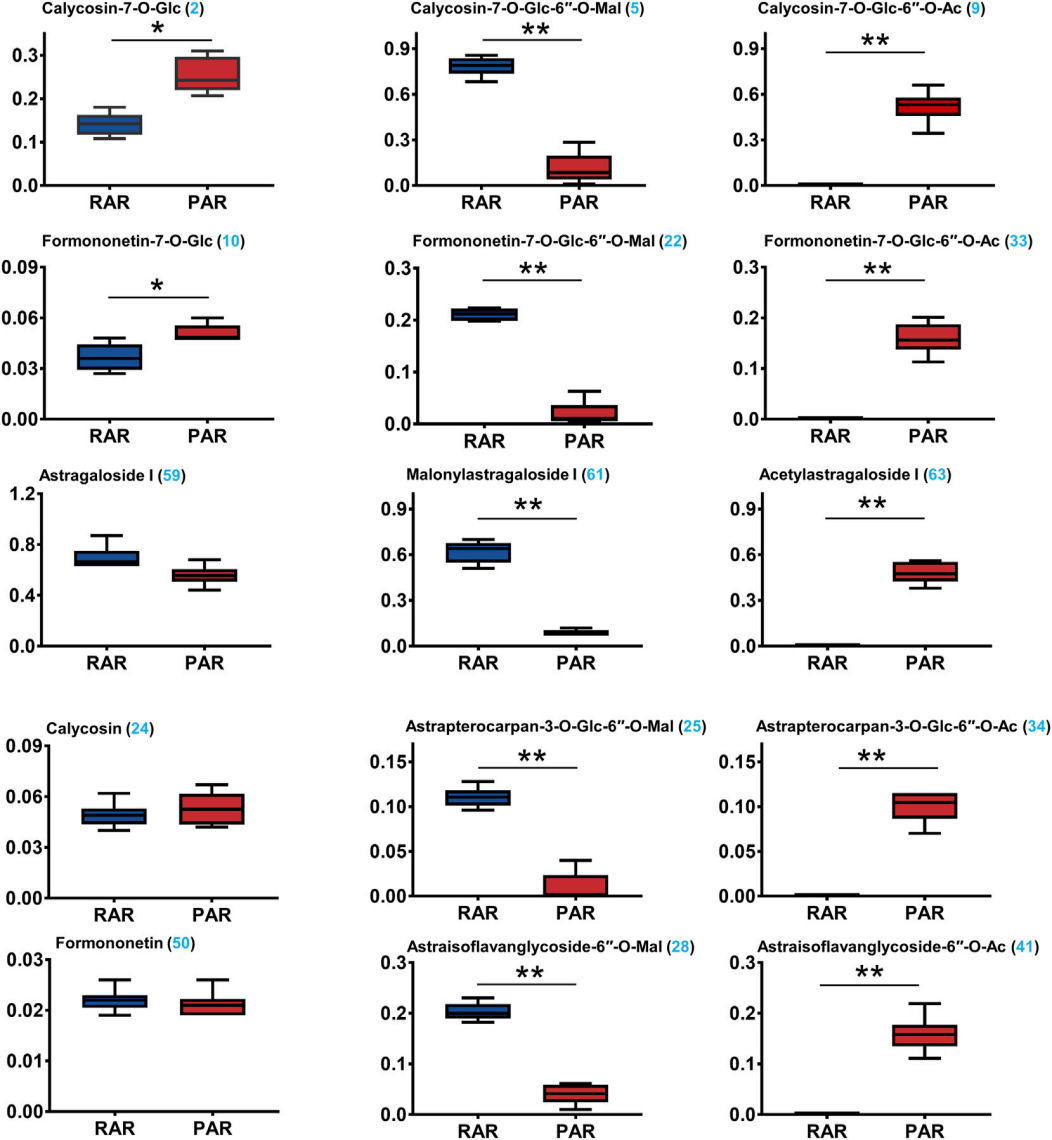
Simultaneous determination of 15 representative ingredients in RAR, and PAR, using high-performance liquid chromatography coupled with diode array detector and evaporative light-scattering detector (HPLC–PDA-ELSD). *p < 0.01; **p < 0.001.

The validated method was applied for simultaneous determination of the 15 selected ingredients in the RAR and PAR samples. The Supplementary Material (Supplementary Figure S6). shows the representative HPLC chromatograms of the 15 representative compounds, RAR and PAR, obtained using a combination of PDA and ELSD detector. The contents of 15 analytes in AR differed greatly Figure 5. Among them, the average contents of the malonyl isoflavonoids and cycloastragenol, calycosin-7*-O-*glycoside-6ʺ*-O-*malonate (**5**), formononetin-7*-O-*glycoside-6ʺ*-O-*malonate (**22**), astrapterocarpan-3*-O-*glycoside-6′*-O-*malonate (**25**), astraisoflavanglycoside-6ʺ*-O-*malonate (**28**), and malonylastragaloside I (**61**) in RAR were 0.748 ± 0.63, 0.211 ± 0.012, 0.203 ± 0.017, 0.110 ± 0.011, and 0.620 ± 0.073 mg/g, respectively, which were respectively approximately 6.8, 10.4, 5.1, 11.5, and 7.0 times higher than those of PAR. Conversely, the corresponding acetyl isoflavonoids/cycloastragenol contents in PAR were 0.519 ± 0.103, 0.159 ± 0.031, 0.159 ± 0.035, 0.100 ± 0.017, and 0.480 ± 0.068 mg/g, respectively, while the contents in RAR being less than the lower detection limit, suggesting that acetyl isoflavonoids and acetyl cycloastragenols in the RAR were converted from the related malonates. In addition, two isoflavonoids glycoside calycosin-7*-O-*glycoside and formononetin-7*-O-*glucoside were more abundant in PAR than in RAR. The above 13 compounds with significant variations (**p < 0.01, ***p < 0.001) between the two ARs were consistent with those screened using non-targeted metabolomics. Of note, astragaloside I (0.697 ± 0.091 and 0.557 ± 0.078 mg/g) and two typical aglycones calycosin (0.049 ± 0.007 and 0.053 ± 0.009 mg/g) and formononetin (0.022 ± 0.002 and 0.021 ± 0.02 mg/g) exhibited no significant difference between RAR and PAR. This was also supported by the non-targeted metabolomic screening. Thus, HPLC–PDA-ELSD quantitative analysis verified differential metabolites between the RAR and PAR, indicating that the LC–QTOF/MS-based comparative non-targeted metabolomics method was reliable.

### General Procedure for the Conversion of Typical Malonyl Compounds

As mentioned above, we speculated that malonyl isoflavonoids/cycloastragenols were converted into the corresponding acetyl derivatives or/and glycosides after the roasting processing of RAR. To prove this hypothesis, chemical conversion experiments were performed on malonyl isoflavonoids/cycloastragenol for characterization of the transformation mechanism Supplementary Material (Supplementary Figure S7).

**FIGURE 6:**
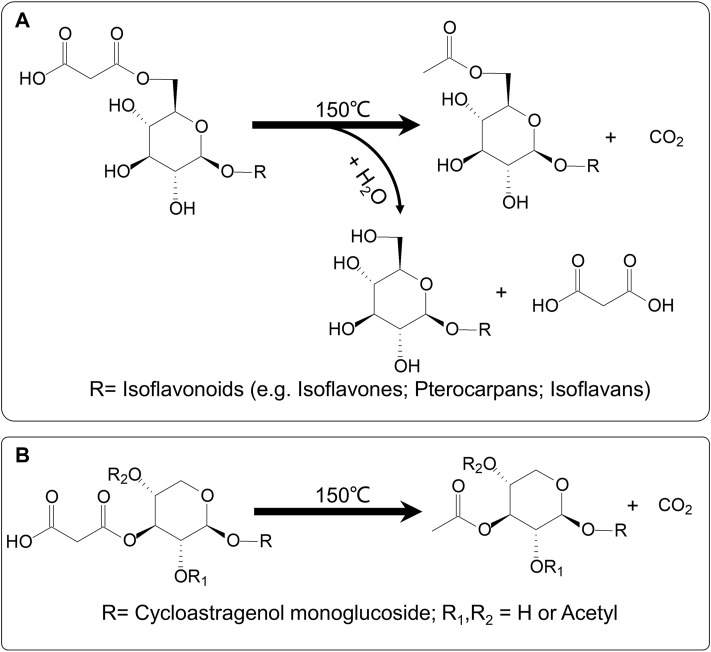
Possible mechanism of chemical transformations during roasting process of AR. **(A)** Malonyl isoflavonoids (e.g., isoflavones, pterocarpans, and isoflavans) could be converted to corresponding acetyl isoflavonoids (major) and glycosides (minor). **(B)** Malonyl cycloastragenols might be transformed into the related acetyl compound only.

By comparing the peak areas of individual compounds, four malonyl isoflavonoids, calycosin-7*-O-*glucoside-6ʺ*-O-*malonate, formononetin-7*-O-*glucoside-6ʺ*-O-*malonate, astraptero-carpan-3*-O-*glucoside-6′*-O-*malonate, and astraisoflavanglycoside-6ʺ*-O-*malonate, significantly decreased during the 30 min heating at 150 ± 10°C, whereas the corresponding acetyl isoflavonoid compounds showed a remarkable improvement. Additionally, the corresponding isoflavonoid glycosides showed a tendency to increase, but the upswing was much lower than that of acetyl isoflavonoids, indicating the occurrence of chemical conversion from malonyl isoflavonoids to related acetyl isoflavonoids (major) and glycosides (minor) under the current roasting conditions. Remarkably, within the timeframe of the conversion experiment, we saw no detectable conversion from the malonylastragaloside I to the astragaloside I form. Indeed, malonylastragaloside I only transformed into a related acetyl compound under the roasting conditions. These results were consistent with that in the PAR formation process, further confirming the mechanism of chemical conversions of the AR as a result of the roasting process (Figure 6).

## Discussion

In this study, an efficient non-targeted RRLC-Q/TOF-MS-based metabolomics method for rapid analysis of RAR and PAR was established. A total of 63 compounds composed of isoflavonoids (e.g., isoflavones, pterocarpans, and isoflavans) and triterpene saponins (e.g., cycloastragenols and oleananes) were identified or tentatively identified by reference substances and their characteristic fragmentation spectra. Multivariate analysis (PCA, OPLS-DA), as well as heatmap and hierarchical clustering analysis, allowed us to identify 29 differential components between the two types of AR. Among them, malonyl isoflavonoids and cycloastragenols were found to be significantly higher in RAR, while corresponding acetyl isoflavonoids or cycloastragenols and related isoflavonoid glycosides were markedly higher in PAR. This result was further supported by quantitative analysis of representative ingredients. Considering that the chemical standards are generally not easy to obtain, this powerful method may be effectively used for the quality evaluation and discrimination of RAR and PAR. It is well known that the malonyl metabolites are widely spread and highly contained in natural plants, and their content may easily change for the instability of malonate. Xiao et al. (2014) found that the content of calycosin-7*-O-*glucoside-6ʺ*-O-*malonate in AR dramatically decreased, whereas calycosin-7*-O-*glucoside increased after being processed. It suggests that a heating procedure during roasting eliminated the malonyl moiety and caused a reduction of the content of calycosin-7*-O-*glucoside-6*-O-*malonate. Our previous research also indicated that AR malonyl isoflavonoids could be hydrolyzed to related isoflavonoid glycosides under elevated solution temperature condition (Zheng et al., 2019). In addition, there was evidence in the literature suggesting that isoflavonoids in AR could have a chemical reaction with glucose under the high temperature and acidic conditions, resulting in dramatically elevated corresponding isoflavonoid glycoside (Xing et al., 2018). However, the results of the present study are completely different from those of prior work. Using a non-targeted metabolomics method combined with HPLC–PDA-ELSD quantitative determination and conversion experiment allowed us to capture the chemical transformations from malonyl isoflavonoids to corresponding acetyl and glycoside compounds during the AR process. Interestingly, under the same conditions, malonyl cycloastragenol type saponins were converted into acetyl compounds without removing malonyl to produce corresponding glycosides. This result may be explained in terms of the structural similarities and differences between the two types of chemical structures. It was suggested that during the roasting procedure, malonyl preferentially loses CO_2_ to form acetyl because of the high temperature and lack of sufficient H_2_O for hydrolysis reaction. Another reason could principally be assigned to the linkage position of malonyl unit to the sugar. In cycloastragenol-type saponins, the malonyl group is likely linked to the 2′, or 3′, or 4′-position of the xylosyl residue, leading to the higher steric hindrance that connects the 6′′ -position of the glucopyranosyl residue in malonyl isoflavonoids.

Traditionally, PAR has been utilized instead of AR to achieve less side effects but improve tonic effects (Huang et al., 2020). In general, the underlying mechanism of variations in clinical efficacy is related to alterations in chemical composition. Therefore, the study of the chemical differences between the RAR and PAR may be helpful to reveal the material basis for the improved in tonic effects of PAR. The previous research (Li et al., 2020) demonstrated that the Qi-tonifying effects of PAR were related to the isoflavonoids and cycloastragenols by mediating immune function. Our study found that malonyl isoflavonoids and cycloastragenols in RAR converted to corresponding acetyl isoflavonoids, cycloastragenols, and isoflavonoid glycosides during roasting, resulting in the high content of acetyl metabolites in PAR. Interestingly, acetyl compounds hold better bioavailability than the malonates because the malonyl moieties are more resistant to intestinal β-glucosidase relative to their simple glucoside counterparts (Hostetler, et al., 2012). Additionally, the acetyl compounds may be converted to deacetyl metabolites and acetate by acetyl esterase from human intestinal microbiome (Zhou et al., 2020). Some recent investigations proved that acetates could contribute to regulation of the host immune system through activation of the immunodeficiency (IMD) pathway (Jugder et al., 2021), and increasing the production of IgA in the colon (Takeuchi et al., 2021). Hence, the transformations of chemical composition from malonates to acetyl compounds during the processing may be related to the enhancement of PAR on the Qi-tonifying effect. Additionally, the concentrations of active compounds and their oral bioavailability were increased after the roasting process (Dai et al., 2020), suggesting the process may play a dual role in enhancing the efficacy. Using *in vivo*/*in vitro* biological activity evaluation and other omics technologies, such as metabolomics and proteomics may possibly clarify the tonic effects of PAR through structural transformation of acetyl isoflavonoids/cycloastragenols. This is a potential direction of research that we will explore in the future. It is worth noting that Maillard reaction should occur during processing (Wong et al., 2018; Ko et al., 2020). However, nearly no relevant compounds were observed. The reason is mainly attributed to thermal instability (Fay and Brevard, 2004). In the detection process of QTOF/MS, the sample should be volatilized at high temperature to form steam, which is furtherly fed into the ion source for ionization. The volatilization treatment process may result in the loss of volatile components, so small furfural compounds cannot be observed in QTOF/MS. What’s more, another type of Maillard reaction products is high molecular weight polymerized brown pigments called melanoidins. In general, the composition and structure of these melanoidin components are very complicated, which were not identified successfully during the screening for differential metabolites. Next, we will attempt to adopt GC-MS to analysis this kind of components in the future.

## Data Availability

The original contributions presented in the study are included in the article/Supplementary Material, further inquiries can be directed to the corresponding author.
