# Supplementary material for: RRLC-QTOF/MS-Based Metabolomics Reveal the Mechanism of Chemical Variations and Transformations of Astragali Radix as a Result of the Roasting Process
Source: Front Chem. 2022 May 5;10:903168. doi: 10.3389/fchem.2022.903168 (PMC9117700; doi:10.3389/fchem.2022.903168)
Supplement: Supplementary file 1 [file DataSheet1.DOCX]

Supplementary Material

**Table S1.** Summary for the tested samples of Astragali Radix

| Sample number | Herbarium NO. | Collected area | Original plant | Data collected |
| --- | --- | --- | --- | --- |
| AR-1 | 190901 | Gu yang, neimenggu province | *A.membraanaceus* var*. mongholicus* | September 2019 |
| AR-2 | 190902 | Gu yang, neimenggu province | *A.membraanaceus* var*. mongholicus* | September 2019 |
| AR-3 | 190903 | Gu yang, neimenggu province | *A.membraanaceus* var*. mongholicus* | September 2019 |
| AR-4 | 191001 | Hun yuan, shanxi province | *A.membraanaceus* var*. mongholicus* | October 2019 |
| AR-5 | 191002 | Hun yuan, shanxi province | *A.membraanaceus* var*. mongholicus* | October 2019 |
| AR-6 | 191003 | Hun yuan, shanxi province | *A.membraanaceus* var*. mongholicus* | October 2019 |

**Table S2.** RSD of retention time of the 14 components in the RAR and PAR

| **No.** | **Retention time (min)** | | | | | | | | | | | | | **RSD**  **(%)** |
| --- | --- | --- | --- | --- | --- | --- | --- | --- | --- | --- | --- | --- | --- | --- |
|  | **Reference** | **RAR1** | **RAR2** | **RAR3** | **RAR4** | **RAR5** | **RAR6** | **PAR1** | **PAR2** | **PAR3** | **PAR4** | **PAR5** | **PAR6** |  |
| **2** | 12.94 | 12.944 | 12.887 | 12.864 | 12.906 | 12.934 | 12.881 | 12.882 | 12.879 | 12.894 | 12.893 | 12.911 | 12.859 | 0.20 |
| **5** | 18.58 | 18.587 | 18.519 | 18.506 | 18.573 | 18.586 | 18.526 | 18.544 | 18.558 | 18.558 | 18.574 | 18.587 | 18.57 | 0.15 |
| **10** | 22.92 | 22.993 | 22.94 | 22.901 | 22.995 | 22.976 | 22.932 | 22.955 | 22.966 | 22.982 | 22.966 | 22.966 | 22.953 | 0.12 |
| **15** | 25.79 | 25.832 | 25.778 | 25.765 | 25.843 | 25.829 | 25.775 | 25.783 | 25.803 | 25.823 | 25.809 | 25.819 | 25.806 | 0.10 |
| **16** | 26.93 | 26.957 | 26.91 | 26.907 | 26.963 | 26.946 | 26.905 | 26.911 | 26.945 | 26.963 | 26.951 | 26.944 | 26.935 | 0.08 |
| **22** | 28.20 | 28.328 | 28.277 | 28.26 | 28.345 | 28.346 | 28.305 | 28.282 | 28.312 | 28.357 | 28.346 | 28.361 | 28.324 | 0.12 |
| **24** | 29.07 | 29.069 | 29.018 | 28.987 | 29.099 | 29.059 | 29.019 | 29.035 | 29.019 | 29.067 | 29.06 | 29.056 | 29.052 | 0.10 |
| **25** | 29.96 | 30.002 | 29.963 | 29.919 | 29.993 | 29.997 | 29.953 | 29.943 | 29.993 | 30.008 | 30.001 | 29.999 | 29.985 | 0.09 |
| **28** | 30.89 | 30.939 | 30.898 | 30.858 | 30.961 | 30.941 | 30.903 | 30.896 | 30.916 | 30.949 | 30.935 | 30.938 | 30.967 | 0.10 |
| **43** | 34.95 | 35.025 | 34.989 | 34.968 | 35.002 | 35.035 | 34.977 | 34.993 | 35.033 | 35.075 | 35.071 | 35.052 | 35.056 | 0.10 |
| **50** | 39.59 | 39.626 | 39.586 | 39.545 | 39.621 | 39.599 | 39.598 | 39.662 | 39.611 | 39.631 | 39.627 | 39.62 | 39.618 | 0.07 |
| **53** | 40.95 | 41.001 | 40.967 | 40.979 | 40.985 | 40.994 | 40.961 | 40.968 | 40.997 | 41.012 | 41.004 | 40.997 | 41.003 | 0.04 |
| **59** | 43.92 | 43.99 | 43.938 | 43.911 | 43.975 | 44.009 | 43.986 | 43.939 | 43.984 | 44.024 | 44.025 | 44.018 | 44.001 | 0.08 |
| **61** | 46.54 | 46.539 | 46.507 | 46.474 | 46.512 | 46.502 | 46.547 | 46.387 | 46.406 | 46.418 | 46.421 | 46.428 | 46.446 | 0.12 |

**Table S3**. ^13^C-NMR（125 MHz）data of acetyl compounds C-1~C-5 in DMSO-d6 (δ in ppm)

| No. | **C-1** | **C-2** | **C-3** | **C-4** | **C-5** | No. | **C-1** | **C-2** | **C-3** | **C-4** | **C-5** |
| --- | --- | --- | --- | --- | --- | --- | --- | --- | --- | --- | --- |
| 1 | - | - | - | - | 34.2 | 26 |  |  |  |  | 27.3 |
| 2 | 153.6 | 153.7 | 66.2 | 69.2 | 29.0 | 27 |  |  |  |  | 27.4 |
| 3 | 123.6 | 123.4 | 39.5 | 31.3 | 88.4 | 28 |  |  |  |  | 28.0 |
| 4 | 174.6 | 174.7 | 78.6 | 29.7 | 41.2 | 29 |  |  |  |  | 15.7 |
| 5 | 127.0 | 127.1 | 132.4 | 130.0 | 51.5 | 30 |  |  |  |  | 19.7 |
| 6 | 115.5 | 115.4 | 110.9 | 108.7 | 78.3 | 1′ | 99.7 | 99.7 | 100.3 | 100.3 | 102.3 |
| 7 | 161.2 | 161.2 | 158.7 | 156.4 | 33.8 | 2′ | 76.2 | 76.2 | 76.8 | 76.4 | 71.4 |
| 8 | 103.4 | 103.6 | 104.5 | 103.8 | 45.5 | 3′ | 73.1 | 73.0 | 73.6 | 73.2 | 71.7 |
| 9 | 156.9 | 157.0 | 156.6 | 154.5 | 20.7 | 4′ | 69.8 | 69.7 | 70.4 | 70.0 | 68.9 |
| 10 | 118.6 | 118.6 | 114.5 | 115.9 | 28.9 | 5′ | 73.8 | 73.8 | 74.1 | 73.6 | 61.6 |
| 11 | 124.4 | 124.0 | 122.0 | 120.8 | 25.6 | 6′ | 63.3 | 64.1 | 63.8 | 63.4 |  |
| 12 | 111.9 | 130.1 | 151.4 | 148.1 | 32.6 | 1′′ | 170.2 |  | 170.7 | 170.3 | 103.7 |
| 13 | 146.1 | 113.7 | 133.8 | 136.1 | 44.4 | 2′′ | 20.7 |  | 20.9 | 20.6 | 74.1 |
| 14 | 147.6 | 159.0 | 153.2 | 151.6 | 45.3 | 3′′ |  |  |  |  | 77.5 |
| 15 | 119.7 | 113.7 | 105.6 | 103.2 | 45.4 | 4′′ |  |  |  |  | 70.7 |
| 16 | 116.4 | 130.1 | 119.2 | 121.4 | 72.4 | 5′′ |  |  |  |  | 76.8 |
| 17 |  |  |  |  | 57.5 | 6′′ |  |  |  |  | 61.4 |
| 18 |  |  |  |  | 20.7 | 14(9)-OCH_3_ | 55.7 | 55.2 | 56.5 | 55.6 |  |
| 19 |  |  |  |  | 31.3 | 13(8)-OCH_3_ | - | - | 60.3 | 60.2 |  |
| 20 |  |  |  |  | 86.4 | 2′-OAc |  |  |  |  | 169.7 |
| 21 |  |  |  |  | 26.7 |  |  |  |  |  | 20.6 |
| 22 |  |  |  |  | 28.4 | 3′-OAc |  |  |  |  | 169.7 |
| 23 |  |  |  |  | 25.6 |  |  |  |  |  | 20.5 |
| 24 |  |  |  |  | 80.7 | 4′-OAc |  |  |  |  | 168.8 |
| 25 |  |  |  |  | 70.3 |  |  |  |  |  | 20.6 |

**Table S4.** Linear Relationships, Limit of Detection (LOD) and Limit of Quantitation (LOQ) of 15 Analytes Obtained Using HPLC-PDA-ELSD

| **Analytes** | **Linearity** | | | **LOD**  **(**μg/mL**)** | **LOQ**  **(**μg/mL**)** |
| --- | --- | --- | --- | --- | --- |
|  | **Calibration Curve** | **R^2^** | **Range** **(**μg/mL**)** |  |  |
| Calycosin-7*-O-*glucoside (**2**) | y = 30960x + 2776.6 | 0.9999 | 1.65-106 | 0.207 | 0.828 |
| Calycosin-7*-O-*glucoside-6''*-O-*malonate (**5**) | y = 30940x + 10896 | 0.9997 | 3.09-198 | 0.387 | 1.55 |
| Calycosin-7*-O-*glucoside -6''*-O-*acetyl (**9**) | y = 37293x + 19734 | 0.9997 | 3.17-203 | 0.396 | 1.59 |
| Calycosin (**24**) | y = 47076x + 2911.8 | 0.9997 | 0.515-33.4 | 0.0645 | 0.258 |
| Formononetin-7*-O-*glucoside (**10**) | y = 36286x + 6624.1 | 0.9996 | 1.78-114 | 0.223 | 0.891 |
| Formononetin-7*-O-*glucoside-6''*-O-*malonate (**22**) | y = 33220x + 4010.7 | 0.9997 | 1.34-86.7 | 0.168 | 0.672 |
| Formononetin-7*-O-*glucoside-6''*-O-*acetyl (**33**) | y = 36866x + 3379.4 | 0.9996 | 1.04-67.5 | 0.131 | 0.523 |
| Formononetin (**50**) | y = 56672x + 695.76 | 0.9997 | 0.312-20.4 | 0.0390 | 0.156 |
| Astrapterocarpan-3*-O-*glucoside-6''*-O-*malonate (**25**) | y = 12705x + 4171.2 | 0.9997 | 3.28-210 | 0.410 | 1.46 |
| Astrapterocarpan-3*-O-*glucoside -6''*-O-*acetyl (**34**) | y = 11961x + 2718.5 | 0.9998 | 3.14-203 | 0.396 | 1.59 |
| Astraisoflavanglycoside-6''*-O-*malonate (**28**) | y = 13467x + 4085.3 | 0.9998 | 3.54-227 | 0.443 | 1.77 |
| Astraisoflavanglycoside-6''*-O-*acetyl (**41**) | y = 12669x + 3564.2 | 0.9997 | 3.12-200 | 0.396 | 1.589 |
| Astragaloside I (**59**) | ln y = 1.8264 ln x + 5.549 | 0.9989 | 20.8-167 | 5.22 | 10.4 |
| Malonylastragaloside I (**61**) | ln y = 1.8407 ln x + 5.6839 | 0.9994 | 19.7-158 | 4.94 | 9.88 |
| Acetylastragaloside I (**63**) | ln y = 1.8232 ln x + 5.7335 | 0.9993 | 23.2-186 | 2.90 | 11.6 |

**Table S5** Precision, repeatability, stability and average recovery of 15 analytes

| Analyte | Precision  (RSD, %) | Repeatability (RSD, %) | Stability  (RSD, %) | Average Recovery (%) /RSD (%) |
| --- | --- | --- | --- | --- |
| Calycosin-7*-O-*Glc (**2**) | 1.63 | 2.03 | 3.72 | 106.2 / 1.96 |
| Calycosin-7*-O-*Glc-6''*-O-*Mal (**5**) | 1.57 | 2.17 | 3.30 | 94.5 / 2.56 |
| Calycosin-7*-O-*Glc-6''*-O-*Ac (**9**) | 0.92 | 1.85 | 1.76 | 99.3 / 2.54 |
| Formononetin-7*-O-*Glc (**10**) | 1.45 | 2.33 | 4.14 | 104.5 / 4.52 |
| Formononetin-7*-O-*Glc-6''*-O-*Mal (**22**) | 1.93 | 2.33 | 2.25 | 95.7 / 3.46 |
| Formononetin-7*-O-*Glc-6''*-O-*Ac (**33**) | 1.08 | 1.45 | 1.96 | 101.2 / 2.40 |
| Calycosin (**24**) | 1.21 | 2.31 | 2.14 | 98.4 / 3.54 |
| Astrapterocarpan-3*-O-*Glc-6''*-O-*Mal (**25**) | 1.85 | 2.12 | 2.74 | 94.4 / 4.83 |
| Astrapterocarpan-3*-O-*Glc-6''*-O-*Ac (**34**) | 1.95 | 2.15 | 1.53 | 93.6 / 4.34 |
| Formononetin (**50**) | 1.54 | 2.38 | 2.01 | 93.3 / 3.74 |
| Astraisoflavanglycoside-6''*-O-*Mal (**28**) | 1.69 | 1.35 | 2.84 | 93.9 / 4.05 |
| Astraisoflavanglycoside-6''*-O-*Ac (**41**) | 0.93 | 1.87 | 2.35 | 97.9 / 3.05 |
| Astragaloside I (**59**) | 1.88 | 1.46 | 2.59 | 104.6 / 4.99 |
| Malonylastragaloside I (**61**) | 2.10 | 2.84 | 3.99 | 92.1 / 6.48 |
| Acetylastragaloside I (**63**) | 1.89 | 2.06 | 1.98 | 97.5 / 3.96 |


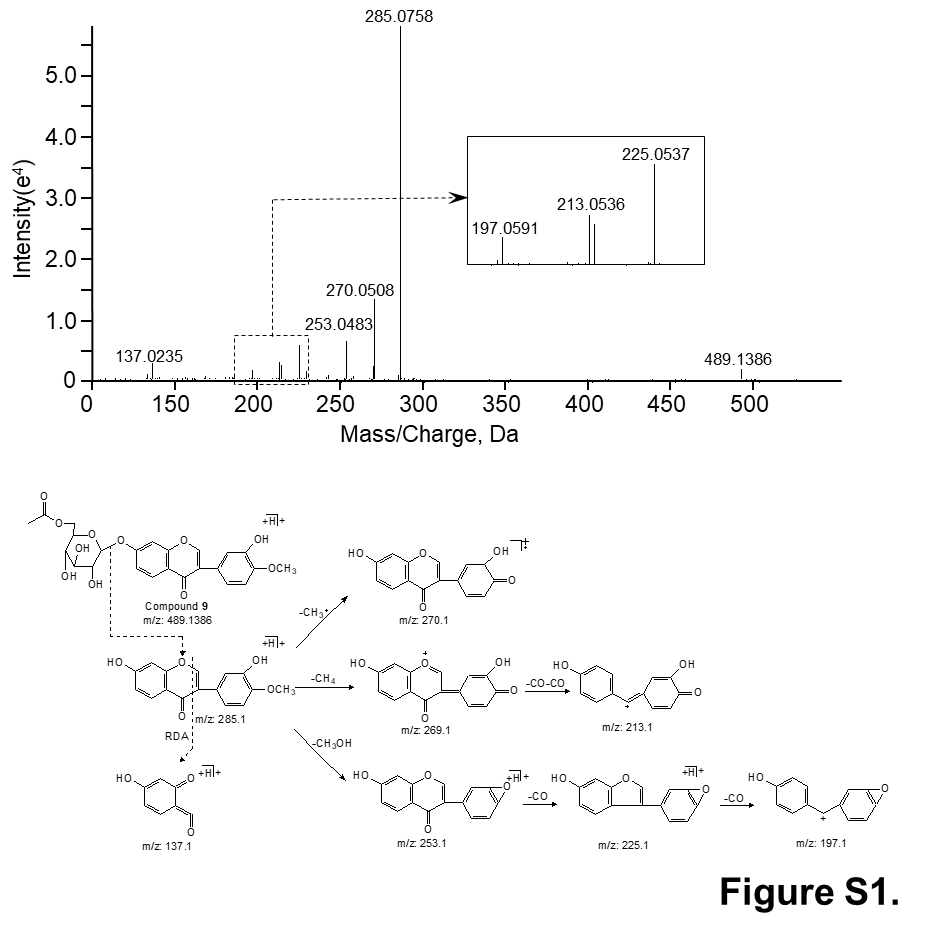


**FIGURE S1.** ESI-Q-TOF/MS (+) spectrum of calycosin-7*-O-*glycoside-6''*-O-*acetyl (9) and its proposed fragmentation pathway.


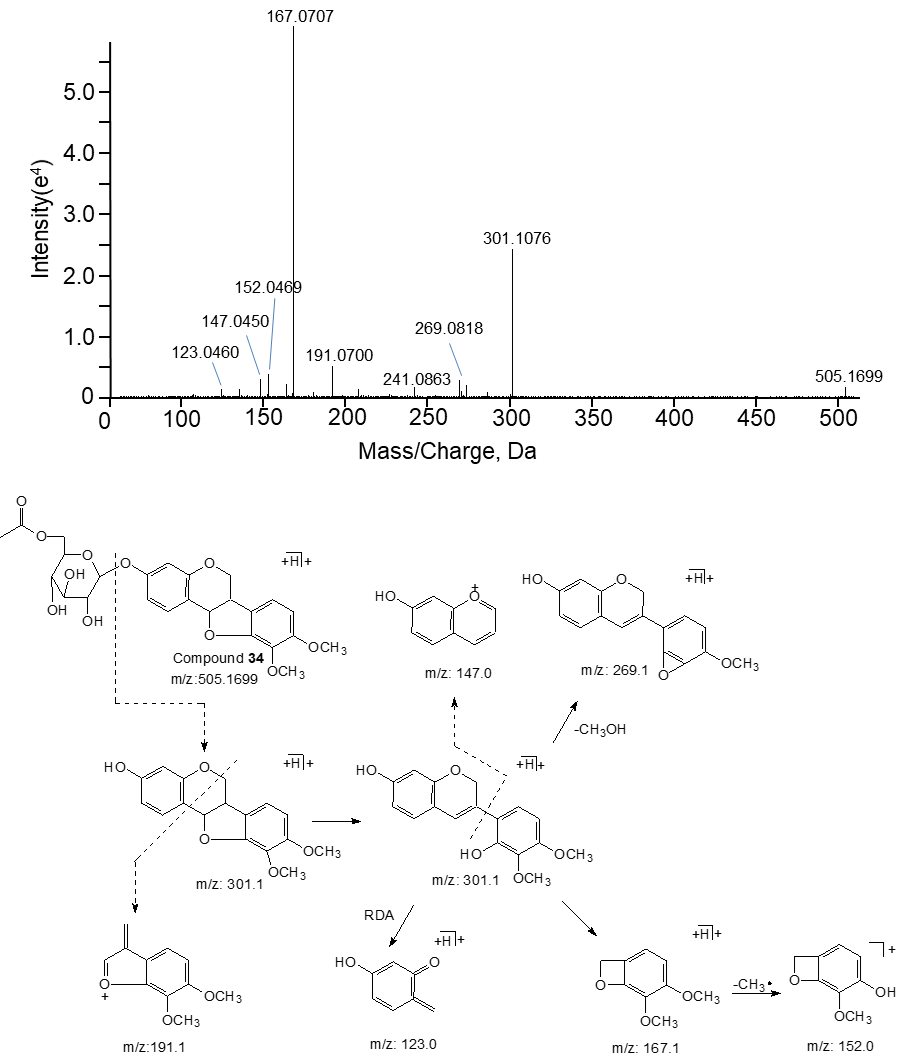


**FIGURE S2.** ESI-Q-TOF/MS (+) spectrum of astrapterocarpan-3*-O-*glycoside-6'*-O-*acetyl (34) and its proposed fragmentation pathway.


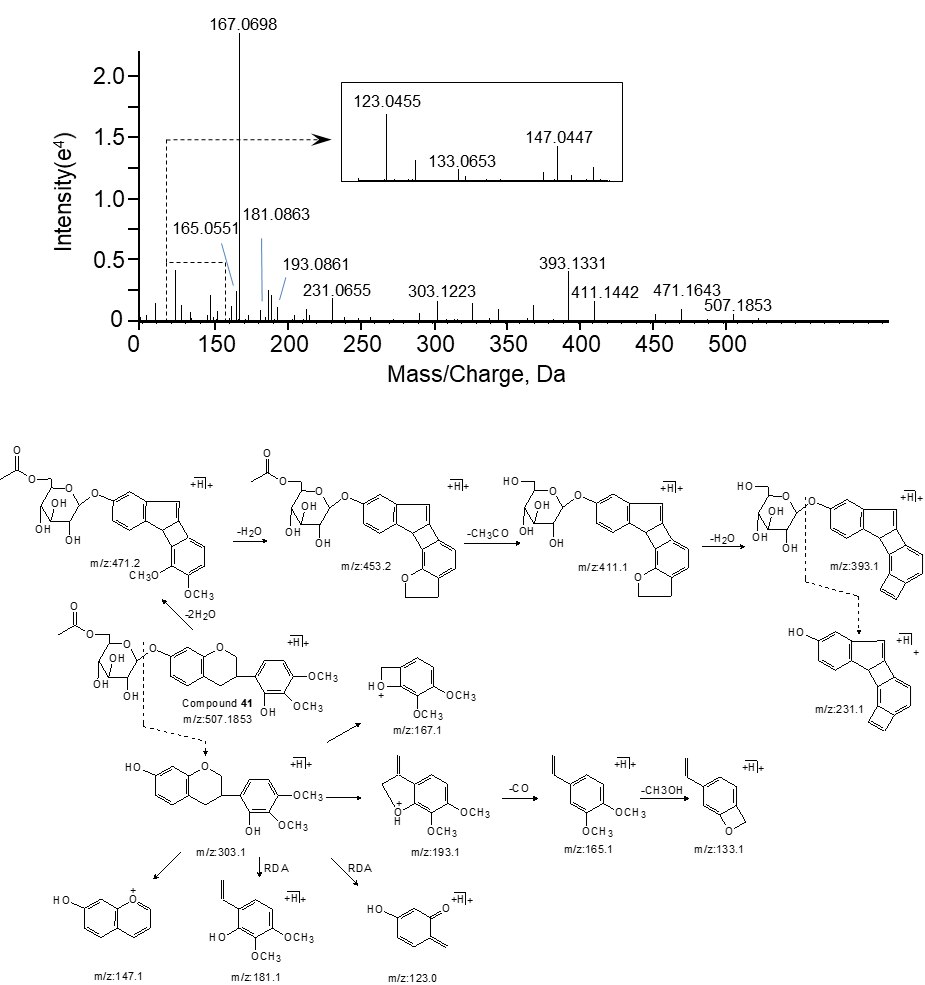


**FIGURE S3.** ESI-Q-TOF/MS (+) spectrum of astraisoflavanglycoside-6''*-O-*acetyl (41) and its proposed fragmentations.


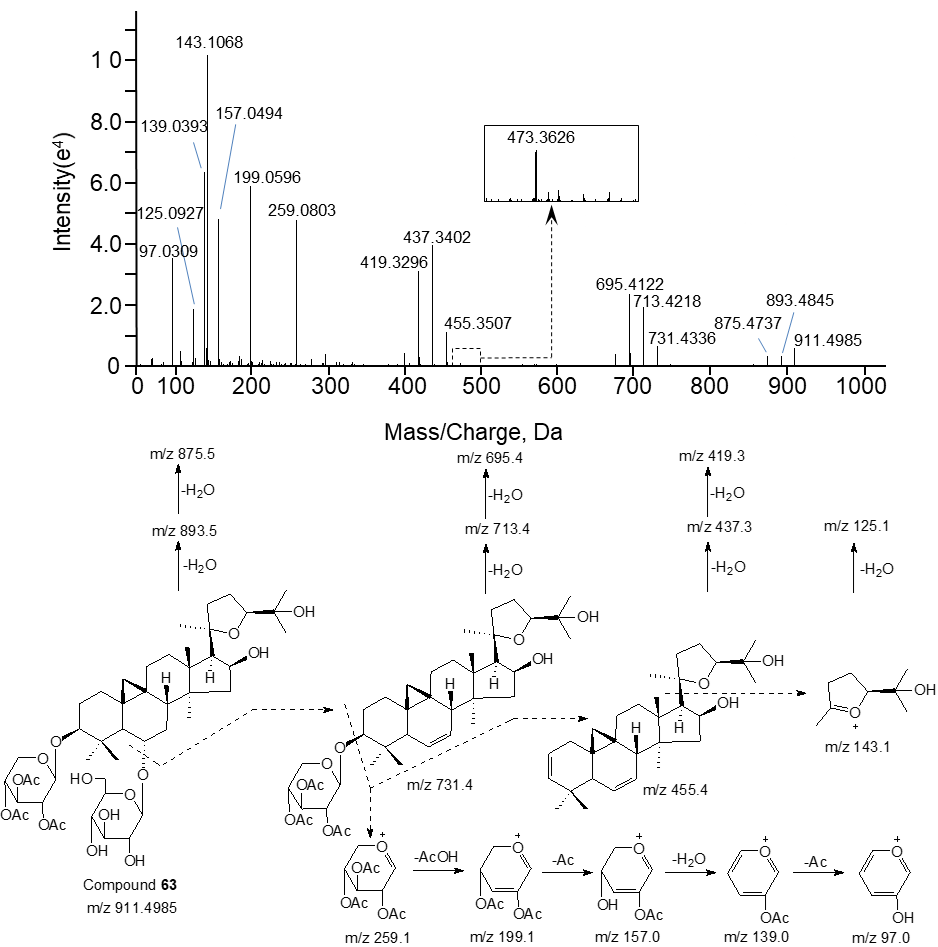


**FIGURE S4.** ESI-Q-TOF/MS (+) spectrum of acetylastragaloside I (63) and its proposed fragmentations.


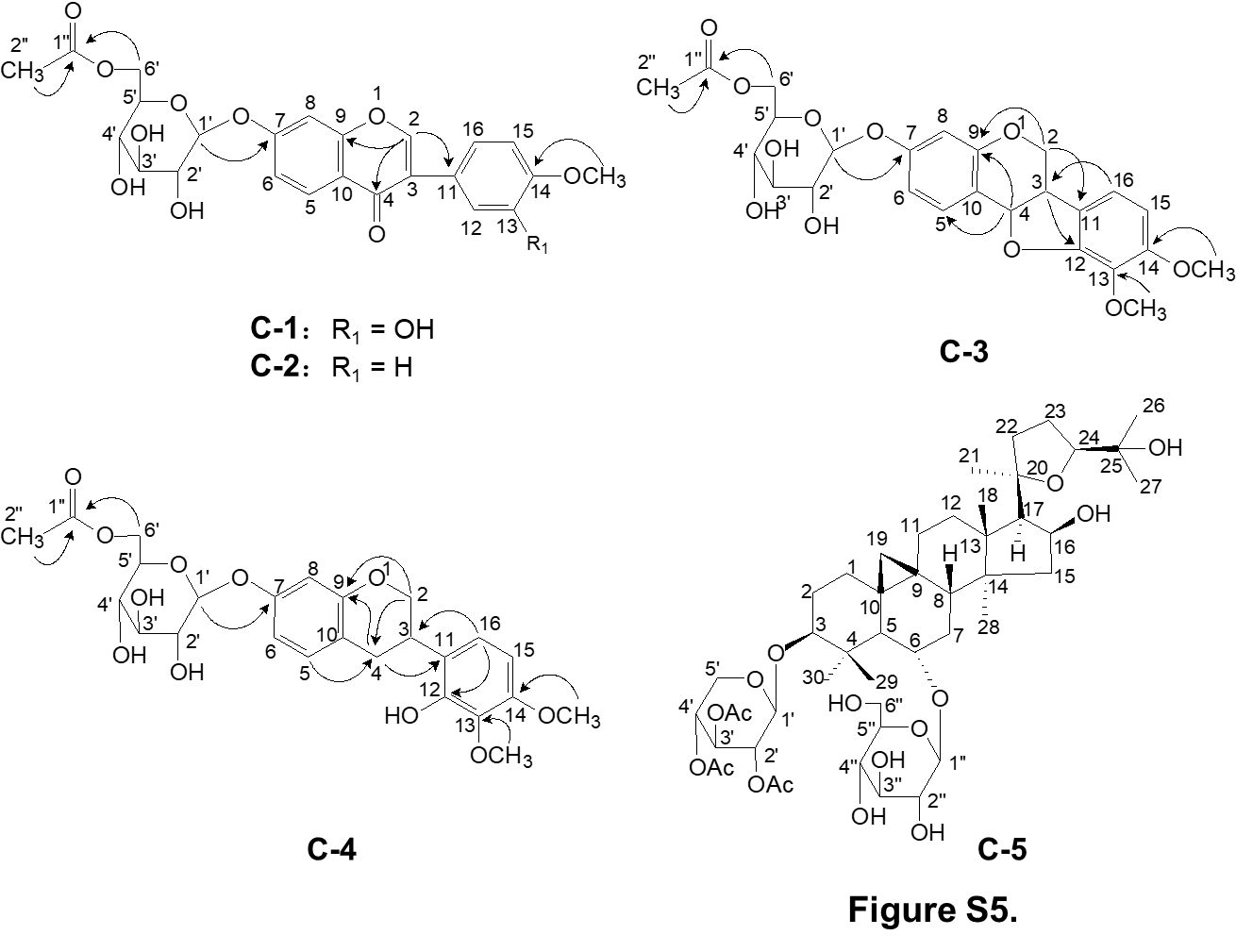


**FIGURE S5.** Chemical structures and key HMBC correlations of the acetyl compounds isolated from PAR.


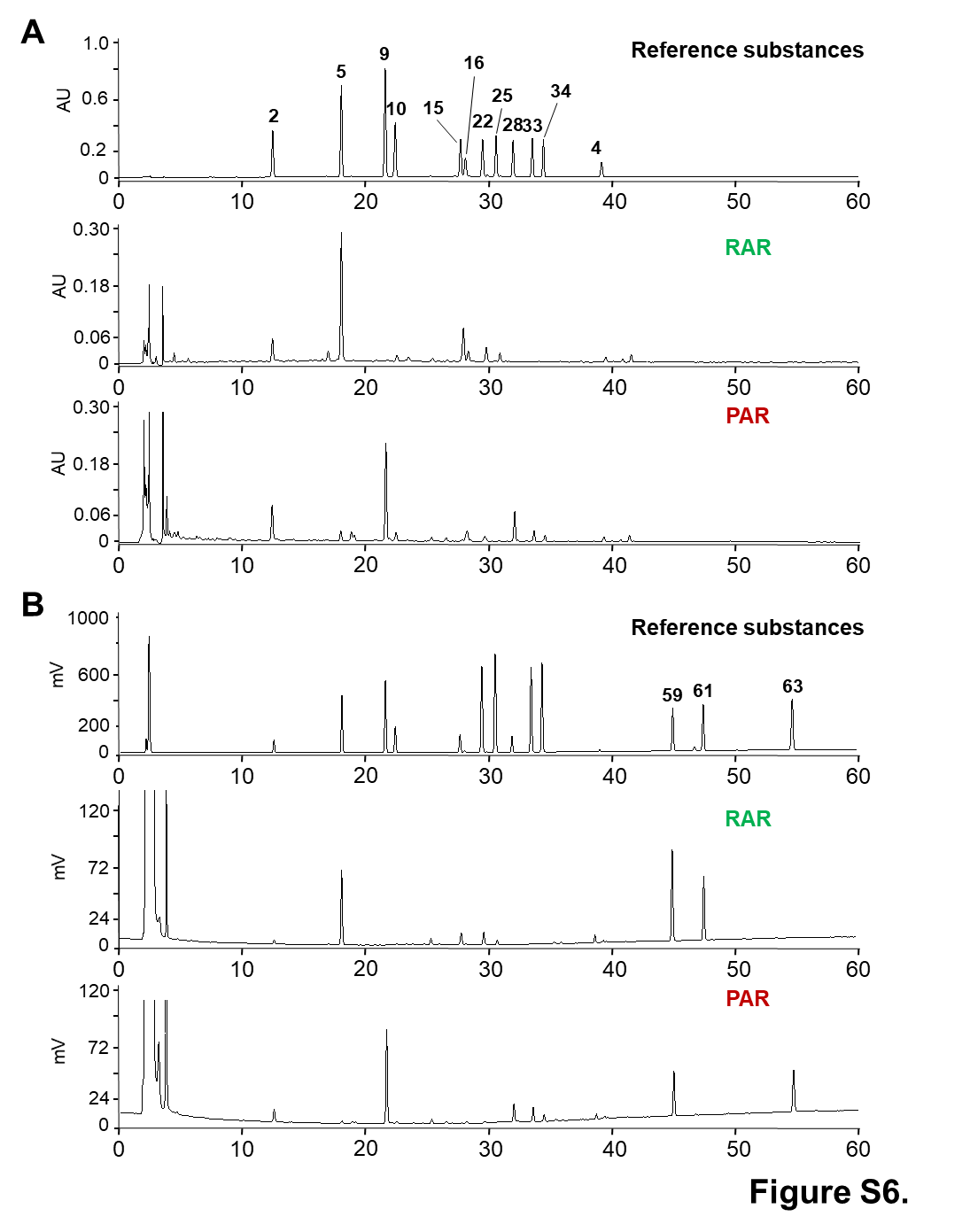


**FIGURE S6.** HPLC chromatograms of determination of 15 representative compounds in raw Astragali Radix (RAR) and Processed Astragali Radix (PAR). (**A**) with PDA detector; (**B**) with ELSD detector.


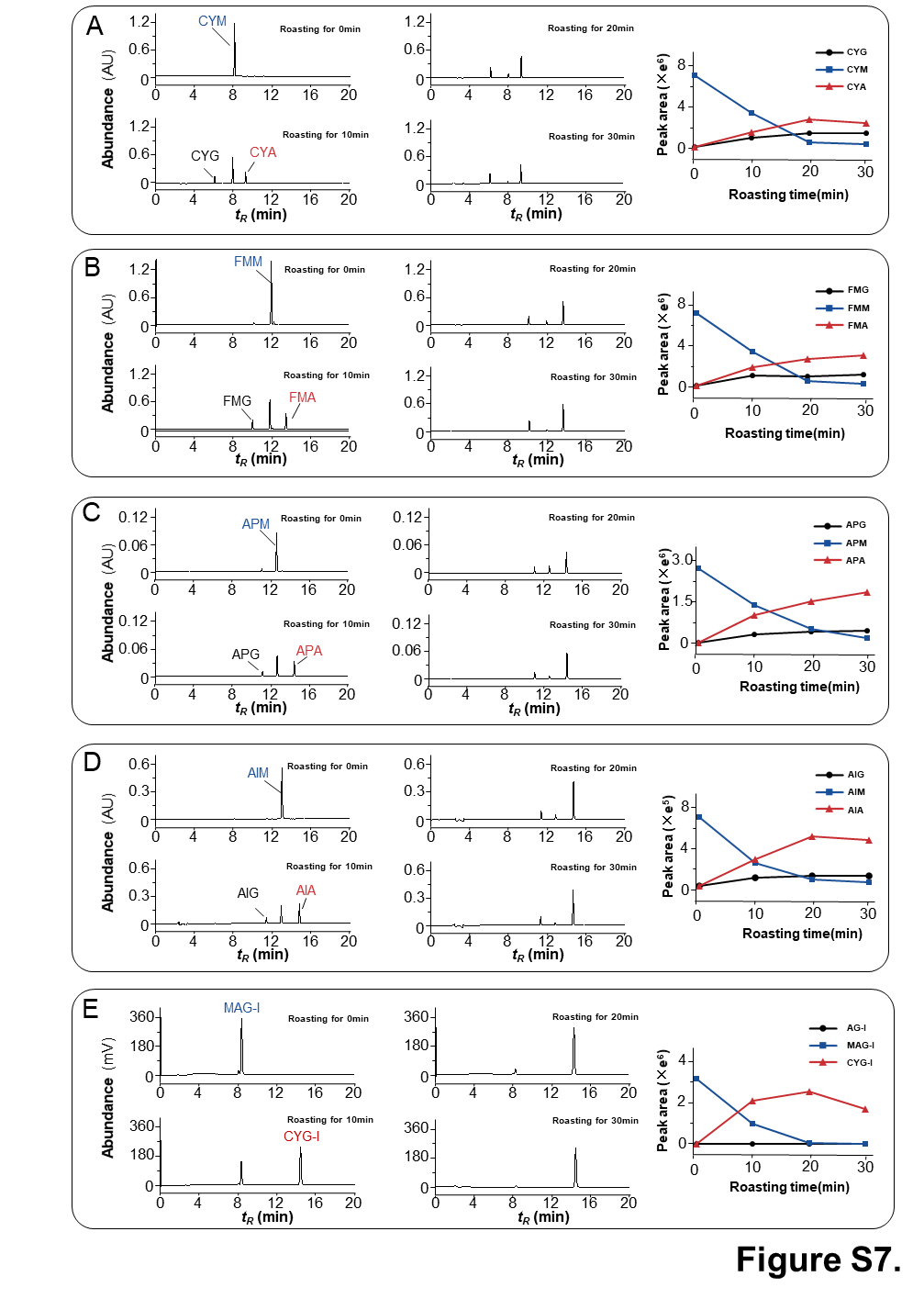


**FIGURE S7.** Chemical conversion of five malonyl isoflavonoids/astragalosides under simulated roasting conditions at 150 ± 10 °C. HPLC charts of chemical transformations during roasting for 30 min are shown (left panel), as well as the amount of metabolite expressed as peak area (right panel). (**A**) Calycosin-7*-O-*glucoside-6ʺ*-O-*malonate (CYM) was converted to calycosin-7*-O-*glycoside-6ʺ*-O-*acetyl (CYA) and calycosin-7*-O-*glycoside (CYG) using HPLC-PDA analysis. (**B**) Formononetin-7*-O-*glycoside-6ʺ*-O-*malonate (FMM) was changed into formononetin-7*-O-*glycoside-6ʺ*-O-*acetyl (FMA) and formononetin-7*-O-*glycoside (FMG) using HPLC-PDA analysis. (**C**) Astrapterocarpan-3*-O-*glucoside-6′*-O-*malonate (APM) was transformed into astrapterocarpan-3*-O-*glucoside-6′*-O-*acetyl (APA) and astrapterocarpan-3*-O-*glucoside (APG) using HPLC-PDA analysis. (**D**) Astraisoflavanglycoside-6ʺ*-O-*malonate (AIM) was converted to astraisoflavanglycoside-6ʺ*-O-* acetyl (AIA) and astraisoflavanglycoside (AIG) using HPLC-PDA analysis. (**E**) Malonylastragaloside I was transformed into astragaloside I using HPLC-ELSD analysis.
